# Zi Shen Decoction Inhibits Growth and Metastasis of Lung Cancer via Regulating the AKT/GSK-3*β*/*β*-Catenin Pathway

**DOI:** 10.1155/2021/6685282

**Published:** 2021-03-09

**Authors:** Yanxia Ma, Yu Liu, Linxin Teng, En Luo, Dekang Liu, Fuqiong Zhou, Kaiyuan Wang, Weiping Chen, Lei Bi

**Affiliations:** ^1^School of Integrated Chinese and Western Medicine, Nanjing University of Chinese Medicine, Nanjing 210023, China; ^2^School of Medicine and Holistic Integrative Medicine, Nanjing University of Chinese Medicine, Nanjing 210023, China

## Abstract

Lung cancer has become the leading cause of cancer-related death worldwide. Oxidative stress plays important roles in the pathogenesis of lung cancer. Many natural products show antioxidative activities in cancer treatment. Zi Shen decoction (ZSD) is a classic prescription for the treatment of lung disease. However, its effect on lung cancer lacks evidence-based efficacy. In this study, we investigated the anticancer effects of ZSD on lung cancer *in vivo* and *in vitro*. Our results showed that oral administration of ZSD suppressed the Lewis lung cancer (LLC) growth in a subcutaneous allograft model and promoted necrosis and inflammatory cell infiltration in the tumor tissues. Furthermore, ZSD not only inhibited tumor cell proliferation and migration but also induced cell apoptosis in lung cancer cells. PI3K/AKT signaling is well characterized in response to oxidative stress. The bioinformatics analysis and western blot assays suggested that ZSD decreased the enzyme activity of PI3K and AKT *in vivo* and *in vitro*. We also found that the AKT/GSK-3*β*/*β*-catenin pathway medicated anticancer effect of ZSD in lung cancer cells. In conclusion, we demonstrate for the first time that ZSD possesses antitumor properties, highlighting its potential use as an alternative strategy or adjuvant treatment for lung cancer therapy.

## 1. Introduction

Lung cancer has been ranked first in morbidity and mortality among all malignant cancers worldwide. According to global cancer statistics, there were 2 million newly diagnosed cases and 1.7 million death cases in 2018 [1, 2]. 80% of lung cancer patients are inoperable at the time of diagnoses. Although chemotherapy and molecular targeted therapy are highly recommended, the efficacy of these treatments is still limited by severe adverse effects and acquired chemoresistance [3]. Therefore, there is an urgent need to identify novel efficient agents for lung cancer treatment. During recent decades, there has been increasingly more attention in natural compounds and traditional Chinese medicine (TCM) for cancer prevention and treatment. Compared with chemotherapy and molecular targeted therapy, TCM therapy showed a series of advantages in lung cancer treatment, including improving long-term survival, overcoming drug resistance, and alleviating the adverse effects of chemotherapy [4–6].

Zi Shen decoction (ZSD) is a TCM formula derived from Synopsis of Prescriptions of the Golden Chamber (JIN GUI YAO LUE), which is compiled by Zhang Zhongjing in Eastern Han Dynasty in China. ZSD is composed of *Salvia miltiorrhiza* Bge. (danshen, salvia) and *Glycyrrhiza uralensis* Fisch. (gancao, glycyrrhiza) and exhibits a remarkable effect on lung disease such as cough and hemoptysis [7]. However, the activity of ZSD in lung cancer has never been shown. TCM theory holds that the deficiency of vital qi, phlegm, and blood stasis are the main pathogenesis of lung cancer, and ZSD possesses the function of strengthening the spleen and lung to remove phlegm and promote blood circulation to remove stasis. Modern pharmacological studies indicate that both salvia and glycyrrhiza show potent anticancer and antioxidative activities both *in vitro* and *in vivo* with representative components [8–10]. Furthermore, our previous study found that Huoxue Yiqi recipe 2, which consists of salvia, glycyrrhiza, and panax ginseng, played an antilung cancer effect [11]. These research suggested an extensive clinical value of ZSD in lung cancer treatment, which has not been investigated elsewhere.

Oxidative stress is involved in the initiation and progression of lung cancer and plays important roles in the pathogenesis due to the accumulation of reactive oxygen species (ROS) [12]. Excessive ROS may promote cell proliferation and invasiveness and suppress apoptosis in cancer progress via affecting different signaling pathways [13–15]. The AKT/GSK-3*β*/*β*-catenin pathway is the “crosstalk” between the PI3K/AKT and Wnt/*β*-catenin pathways, which is one of the most studied pathways in cancer development. AKT is a pivotal downstream effector of PI3K signal, which can be activated by excessive ROS accumulation or growth factors (EGF, FGF, and VEGF) and cytokine (IL-3, IL-6, and IL-2) stimulation [16–18]. On the state of the Wnt/*β*-catenin pathway, glycogen synthesis kinase-3*β* (GSK-3*β*) can phosphorylate *β*-catenin, leading to the ubiquitination degradation of *β*-catenin. This process can be reversed by AKT-induced phosphorylation of GSK-3*β* at serine 9, causing the accumulation of *β*-catenin in the cytoplasm and migration into the cell nuclei. *β*-Catenin in the cell nucleus interacts with T cell-specific factor (TCF)/lymphoid enhancer-binding factor (LEF) and coactivators to activate transcription of downstream target genes, such as c-Myc, cyclin D1, and E-cadherin, which induces growth and metastasis of cancer cells [19–22]. Therefore, targeting AKT/GSK-3*β*/*β*-catenin is a novel approach in cancer treatment.

Here, the present study identified the compound-target network of ZSD and predicted the potential role in lung cancer treatment via network pharmacology analysis, and then, the anticancer effects and the potential pathway of ZSD against lung cancer were examined *in vitro* and *in vivo*. Our research demonstrated, for the first time, that ZSD possessed therapeutic effect on lung cancer and elucidated that the AKT/GSK-3*β*/*β*-catenin pathway was partially involved in its anticancer effect. Thus, these findings provide a new insight for future lung cancer therapy.

## 2. Materials and Methods

### 2.1. Preparation of ZSD

*Salvia miltiorrhiza* Bge. (Salvia, Batch No. 111117) and *Glycyrrhiza uralensis* Fisch. (glycyrrhiza, Batch No. 180720) were provided by Nantong Sanyue Chinese Herb Pieces Co. Ltd. (Nantong, China) and identified by Prof. Weiping Chen (Nanjing University of Chinese Medicine, Nanjing, China). Salvia (125 g) and glycyrrhiza (47 g) were decocted with 1 L of distilled water for 2 h. The water extract was concentrated to 1 mg/mL. The supernatant was taken after centrifugation, filtration with 0.22 *μ*m filter membrane, and sterilization and stored at -20°C for long-term use. High-performance liquid chromatograph (HPLC, Thermo, MA, United States) with ODS-SP C18 column (250 mm × 4.6 mm, 5 *μ*m) was applied to analyze the chemical components of ZSD aqueous extract. The mobile phase is deionized water with 0.2% formic acid (A) and acetonitrile with 0.2% formic acid (B) and gradient elution program was as follows: 0-14 min, 2-10% B (*v*/*v*); 14-29 min, 10-12% B; 29-52 min, 12-15% B; 52-65 min, 15-20% B; 65-70 min, 20-30% B; 70-80 min, 30-45% B; and 80-88 min, 45%-88% B. The HPLC results are shown in Figure S1 and a total of 4 components in ZSD were detected, namely, salvianic acid A, liquiritin, salvianolic acid B, and salvianolic acid A, and the concentrations were 2.33 mg/mL, 0.14 mg/mL, 4.48 mg/mL, and 0.83 mg/mL, respectively.

### 2.2. Compound Collection of ZSD

All of the chemical ingredients of salvia and glycyrrhiza were collected from the Traditional Chinese Medicine Systems Pharmacology database (http://lsp.nwu.edu.cn/tcmsp.php). Oral bioavailability (OB) value represents the efficiency of drug delivery in pharmacodynamics. Drug-like (DL) is an evaluating indicator in drug design. Herein, we set OB ≥ 30% and DL ≥ 0.17 as the inclusion criteria for the preliminary screening of the collected compounds.

### 2.3. Corresponding Target Intersection of ZSD

The potential targets of the active compounds in ZSD were searched from 3 databases, namely, Similarity Ensemble Approach (SEA, http://sea.bkslab.org/), Bioinformatics Analysis Tool for Molecular Mechanism of Traditional Chinese Medicine (BATMAN-TCM, http://bionet.ncpsb.org/batman-tcm/), and the Swiss target prediction (SIB, http://www.swisstargetprediction.ch/).

### 2.4. Network Construction and Gene Ontology (GO) and KEGG Pathway Enrichment Analysis

The common putative targets from 3 databases were collected. The compound-target network was imported into Cytoscape to show the association between the targets and the active compounds. Then, the gene ontology (GO) function enrichment and KEGG pathway enrichment of those targets obtained from databases were carried out by clusterProfiler, and the visualization analysis was conducted by topGO. In the programming language, *p* value cutoff = 0.05.

### 2.5. Molecular Docking

Key targets involved in the non-small-cell lung cancer pathway and the corresponding compounds were obtained. Molecular docking was performed using the LibDock module of Discovery Studio 4.0. The docking results are assessed by the LibDock score. The higher LibDock score means the higher activity of the predicted component binding to the target.

### 2.6. Chemicals and Antibodies

Antibodies against total AKT and p-AKT, total GSK-3*β* and p-GSK-3*β*, *β*-catenin, cleaved caspase-3, and GAPDH were purchased from Cell Signaling Technology (Danvers, MA, USA). Antibodies specifically against *β*-actin and Bax and Bcl-2 were purchased from Abcam (Cambridge, UK). Dulbecco's modified Eagle medium (DMEM) and fetal bovine serum (FBS) were purchased from Sigma-Aldrich (St. Louis, MO, USA). Roswell Park Memorial Institute (RPMI-1640) was purchased from HyClone (Logan, UT, USA). Dimethyl sulfoxide (DMSO), trypsin, penicillin-streptomycin solution, and 3-(4,5-dimethylthiazol-2-yl)-2,5-diphenyl-2H-tetrazolium bromide (MTT) dye were from Solarbio (Beijing, China). RNAiso Plus, PrimeScript™ RT Master Mix (Perfect Real Time), and SYBR Green® Premix Ex Taq™ II (Tli RNaseH Plus) were purchased from Takara (Kusatsu, Shiga, Japan). Phenylmethanesulfonyl fluoride (PMSF), protease inhibitor cocktail, phosphatase inhibitor cocktail, and lane marker loading buffer were from Beyotime (Shanghai, China). Glycine, Tris Base, multicolor protein marker, Tween 20, and sodium dodecyl sulfate-polyacrylamide gel electrophoresis (SDS-PAGE) were purchased from Solarbio (Beijing, China). Polyvinylidene fluoride membranes (PVDF) were purchased from Millipore (Millipore, Bedford, MA, USA), and the ECL reagent was purchased from ComWin (Beijing, China). BSA was purchased from Aladdin (Shanghai, China). SC-79 was from MedChemExpress (Shanghai, China). All buffers and solutions were prepared with water obtained from the Milli-Q Synthesis water purification system (Millipore, MA, USA).

### 2.7. *In Vivo* Tumor Growth

Four-week-old male C57BL/6 mice were purchased from Zhao Yan (Suzhou) New Drug Research Center Co., Ltd. (Suzhou, China). Mice were maintained in specific pathogen-free (SPF) environment with standard rodent chow and water and light-dark cycles every 12 h with constant room temperature before the experiment. LLC cells (3 × 10^6^ cells in 100 *μ*L PBS) were subcutaneously injected into the skin over the right shoulders of the mice. The mice were randomly divided into four groups (*n* = 6 per group): model group (normal saline (NS): 0.2 mL/10 g/d); HSOL group (hui-sheng oral liquid: 0.585 mg/kg/d); ZSD low-dose group (ZSDL: 11.18 g/kg/d); and ZSD high-dose group (ZSDH: 44.7 g/kg/d). Normal saline, ZSD, and HSOL were administered to mice every day for 21 days via intragastric administration. The body weight of mice and volume of the tumors were measured every 3 days. Finally, the animals were sacrificed with cervical dislocation and tumors were harvested for further analyses. All animal experiments were approved by the Institutional Animal Care and Use Committee of the Nanjing University of Chinese Medicine (201809A020).

### 2.8. Cell Lines and Cell Culture

The human normal lung epithelial cell line (BEAS-2B), human NSCLC cell lines (A549, H1975), and mouse LLC cell line were obtained from Shanghai Institute of Biochemistry and Cell Biology. Human NSCLC cell line (H1299) was purchased from Jiangsu Key GEN Bio TECH Corp. BEAS-2B and A549 cells were cultured in DMEM medium supplement with 10% FBS and 1% PS. H1299 and H1975 cells were cultured in RPMI-1640 medium supplement with 10% FBS and 1% PS. All these cell lines were maintained in a humid atmosphere with 5% CO_2_ at 37°C.

### 2.9. Cell Viability Assay

The variation of cell viability affected by different treatments was measured by MTT assay. Briefly, cells (5000 cells/well) were seeded in a 96-well plate overnight, and then, different doses of ZSD were added into each well. After treatment, 20 *μ*L of MTT (5 mg/mL) solution was added to each well. Afterwards, the 96-well plate was incubated at 37°C for 4 h, and then, formazan crystals were dissolved by 150 *μ*L DMSO. Finally, the absorbance was measured at 490 nm by using a microplate reader. The values acquired from three independent experiments were used to calculate IC50 values using GraphPad Prism 8.

### 2.10. Clone Formation Assay

200 cells/well were seeded to six-well plates and treated with different concentrations of ZSD (0, 0.25, 0.5, 1.0, 2.0, and 4.0 mg/mL). The medium was replaced with fresh culture medium every 3 days. After 2 weeks of culture with drug medium, the colony is clearly visible. Subsequently, the colonies were fixed with methanol, stained with crystal violet, photographed, and counted using the ImageJ software.

### 2.11. Wound-Healing Assay

Wound-healing assay was performed to determine the effect of ZSD on migratory ability of cells. H1299, H1975, A549, and BEAS-2B cells were seeded in 6-well plates with culture medium containing 1 × 10^5^ cells in each well and allowed to grow to 90% density. Cell monolayers were wounded by scratching with a 200 *μ*L pipette tip and then washed lightly with PBS three times. Then, the cells were exposed to various concentrations of ZSD for 48 h. The wound gap was observed and images were obtained at 0 h, 24 h, and 48 h with an inverted fluorescence microscope (×100 magnification), and the average distance of cell migration was measured using ImageJ software.

### 2.12. Transwell Migration Assay and Invasion Assay

Briefly, to measure cell migration, 2 × 10^5^ cells were plated on the upper 24-well transwell chambers (8 *μ*m pore size, Millipore) in 200 *μ*L serum-free media with or without ZSD. The lower chambers were filled with 600 *μ*L medium containing 10% FBS. For the invasion assay, 50 *μ*L of Matrigel (BD Biosciences, Franklin Lakes, NJ, USA) was added to the chambers, and 2 × 10^5^ cells were seeded into the chamber with 100 *μ*L of serum-free medium. Then, the 24-well transwell chambers were incubated at 37°C for 24 h. After incubation, the medium was discarded and cells were removed by a cotton swab softly. Migrated or invaded cells in the lower chamber were fixed with methanol and stained with 0.5% crystal violet. The images were taken with an inverted microscope (×100 magnification), and the number of cells was counted using ImageJ software.

### 2.13. Quantitative Real-Time PCR

Total RNA was isolated from NSCLC cells or mice tumor tissues with RNAiso plus reagent. 500 ng of RNA was converted into cDNA using PrimeScript™ RT Master Mix according to the manufacturer's instructions. SYBR Green® Premix Ex Taq™ II was used for the amplification in the real-time Q-PCR system (Applied Biosystems, Foster City, CA, USA). The cDNA of reverse transcription was used to conduct Human Signal Transduction Pathway Finder RT^2^ Profiler™ PCR Array and qPCR assay. The mRNA levels of PI3K, AKT, GSK-3*β*, *β*-catenin, caspase3, Bax, and *β*-actin in tumor samples and lung cancer cells were accessed. The sequences of these primers are showed in Table S1.

### 2.14. Flow Cytometry Analysis

Cell apoptosis was analyzed by Annexin V-FITC/Propidium Iodide (PI) Apoptosis Detection Kit according to the manufacturer's protocol (Sigma-Aldrich, Sigma Chemical Co., St. Louis, MO, USA). Briefly, H1299 cells were seeded in 6-well plates at a density of 1 × 10^4^ cells/well and then treated with ZSD (4 mg/mL) for 12, 24, and 48 h, respectively, or different doses of ZSD. Then, cells were collected and centrifuged and resuspended in binding buffer. Afterwards, 5 *μ*L Annexin V-FITC and 5 *μ*L PI were added at room temperature for 15 min. Finally, apoptosis cells were analyzed by a flow cytometer (FACSC, BD Instruments Inc., USA) and FlowJo 7.1.0 software (Tree Star, Ashland, OR, USA).

### 2.15. Western Blot

The total protein was extracted from the ZSD-treated H1299 cells and in vivo cancer tissues by using RIPA cell lysis buffer supplemented with PMSF, proteinase, and phosphatase inhibitors. BCA protein assay kit was used to determine the protein concentration according to the manufacturer's instructions. Equal amounts of protein samples (40 *μ*g) were loaded and separated in SDS-PAGE. After transferring onto the PVDF membrane, the proteins were blotted with 5% BSA, followed by probing with primary antibodies and then incubated with goat anti-rabbit or goat anti-mouse secondary antibodies. Finally, the membranes were visualized with enhanced chemiluminescence.

### 2.16. Hematoxylin and Eosin Staining

The tumor tissues were fixed with formalin, dehydrated by ethanol and placed in xylene, and then embedded in paraffin wax and sliced. The sections were baked at 70°C for 4 h, dewaxed, hematoxylin stained for 15 minutes, decolorized with hydrochloric acid alcohol solution for 5 seconds, stained with eosin for 2 minutes, routinely dehydrated, and then mounted in neutral gel. The tumor tissue sections were observed under a microscope.

### 2.17. Statistical Analysis

All statistical analyses were performed using GraphPad Prism 8.0 software. Results are expressed as mean ± SD. Compared to that of the control group, the difference analysis of the treatment groups was accessed by using one-way analysis of variance (ANOVA). The significance of differences is indicated at ^∗^*p* < 0.05, ^∗∗^*p* < 0.01, and ^∗∗∗^*p* < 0.001.

## 3. Results

### 3.1. Network Pharmacology Analysis of ZSD

**Figure 1:**
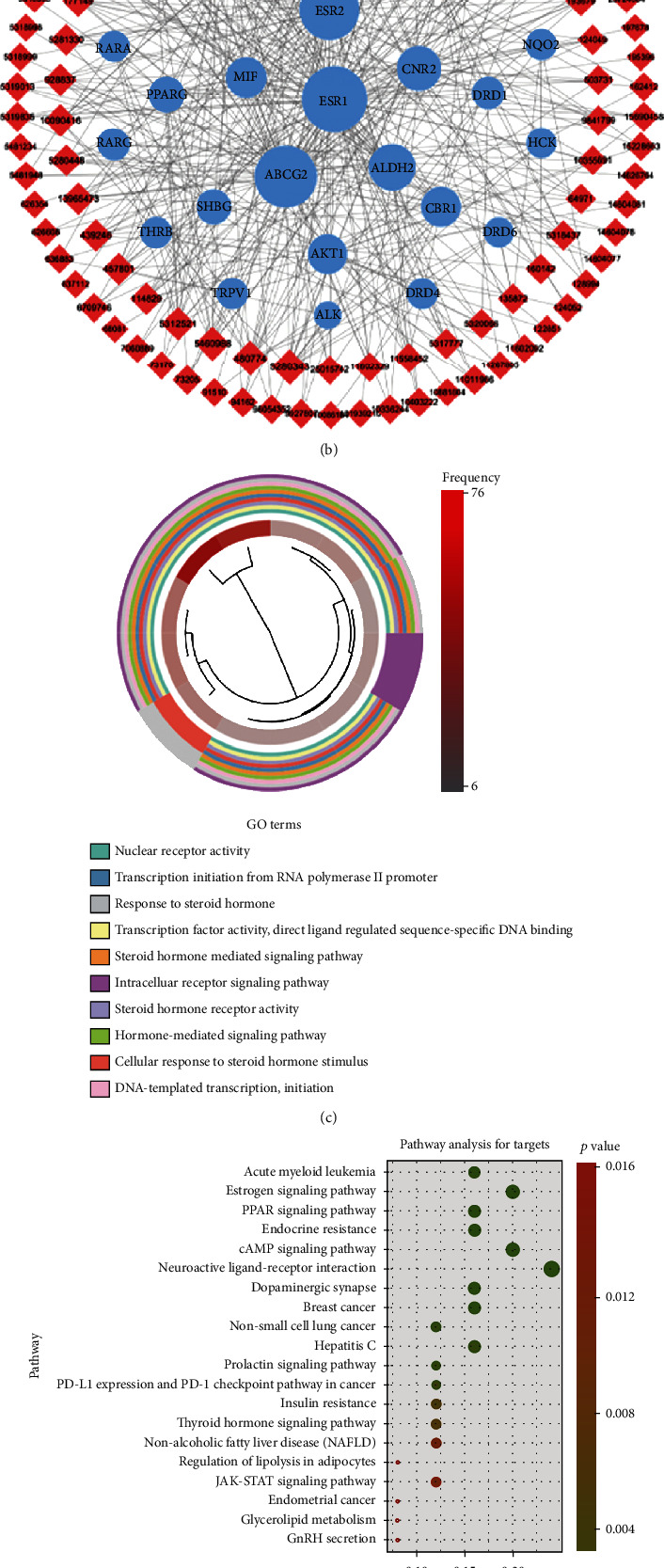
The network pharmacology analysis of ZSD. (a) The flowchart of the network pharmacology. (b) Compound-target network: the red diamond nodes represent compounds of ZSD. The blue circle nodes represent corresponding targets. (c) GO enrichment analysis of the candidate targets of ZSD. (d) KEGG pathway enrichment analysis of candidate targets of ZSD. (e) Molecular docking process of 2-(3,4-dihydroxyphenyl)-5,7-dihydroxy-6-(3-methylbut-2-enyl) chromone-4-one with AKT1. (f) Docking process of quercetin with AKT1. (g) Docking process of quercetin with EGFR. (h) Docking process of quercetin with ALK.

As shown in Figure 1(a), 135 compounds of ZSD satisfying the criteria were retrieved from TCMSP, and the compound information was queried and standardized in PubChem database. All compounds were listed with CAS number or PubChem ID. Then, 589, 210, and 364 targets of all these compounds were collected from BATMAN-TCM, SEA database, and SWISS database, respectively. By intersecting these databases, 28 targets were identified as putative therapeutic targets of ZSD. Then, the compounds and 28 targets were applied to construct the compound-target (C-T) network using Cytoscape 3.6.0 software (Figure 1(b)). The gene ontology (GO) enrichment analysis of the 28 targets was performed to analyze relative biological functions, including biological process, cell components, and molecular functions (Table S2). The top 10 significantly enriched terms were listed based on biological process (Figure 1(c)). The results suggest that these targets may exert therapeutic effects by regulating cell response to steroid, transcription initiation from DNA polymerase II promoter, DNA-templated transcription, and intracellular receptor signaling pathways. Then, pathway enrichment analysis of the 28 targets was performed (Table S3) and the top 20 is shown in Figure 1(d), suggesting that ZSD may interfere with cancer-related pathways, including the acute myeloid leukemia pathway, breast cancer pathway, and non-small-cell lung cancer pathway. Furthermore, the targets in non-small-cell lung cancer pathway were docked with the corresponding compounds and the results showed that 2-(3,4-dihydroxyphenyl)-5,7-dihydroxy-6-(3-methylbut-2-enyl) chromone-4-one and quercetin had good docking ability with AKT1, EGFR, and ALK protein (Figures 1(e)–1(h)). Noteworthily, the consistency of the molecular docking results with the network pharmacology screening results indicated the potential effect of ZSD on lung cancer treatment. To prove if this is the case, we next validated the antitumor effect of ZSD *in vitro* and *in vivo*.

### 3.2. ZSD Suppresses the Tumor Growth of LLC-Allograft Mouse Model

**Figure 2:**
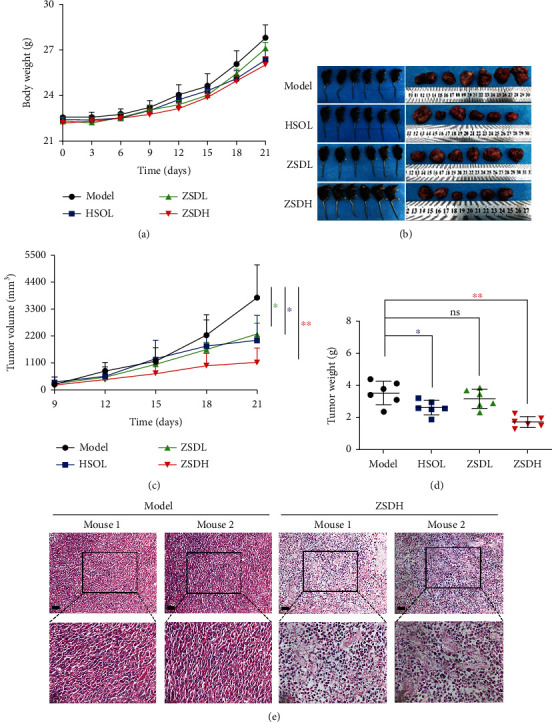
ZSD suppressed growth of LLC-allograft tumors. (a) The body weight of mice in each group. (b) Images of mice injected subcutaneously with LLC cells and tumor tissues from LLC-allograft mice treated with NS, HSOL, and ZSD. (c) Tumor volumes were recorded every two days during the treatment. (d) Mice were sacrificed after 21 days of treatment, and representative tumors and the weight in each group were showed. (e) H&E staining of tumor tissues from the model and high dose of ZSD group. Scale bar: 200 *μ*m.

We first investigated the antitumor effect of ZSD *in vivo* with an LLC-allograft model in C57BL/6 mice as described [23]. Hui-sheng oral liquid (HSOL), which has been used in clinical treatment of primary lung cancer and liver cancer for years, was used as a positive control drug [24, 25]. As shown in Figure 2(a), there was no significant difference in body weight among the model, HSOL, and the ZSD treatment groups, suggesting that ZSD treatment was well tolerated. The representative images of the mice and tumors in each group showed that HSOL and ZSD remarkably suppressed tumor growth in mice after three weeks of treatment (Figures 2(b) and 2(c), *p* < 0.05 and *p* < 0.01). Meanwhile, compared with the model group, the tumor weight in the HSOL and ZSDH groups was significantly reduced but not in the ZSDL group (Figure 2(d)). The H&E staining showed that the ZSDH treatment group exhibited increased inflammatory cell infiltration and necrosis in tumor tissues (Figure 2(e)). Interestingly, we also found that mice in the ZSDH and HSOL groups showed higher thymus index than those in the model group (Figure S2). These data suggest that ZSD could be an effective anticancer drug for lung cancer without serious toxicity.

### 3.3. ZSD Inhibits the Proliferation of Lung Cancer Cells *In Vitro*

**Figure 3:**
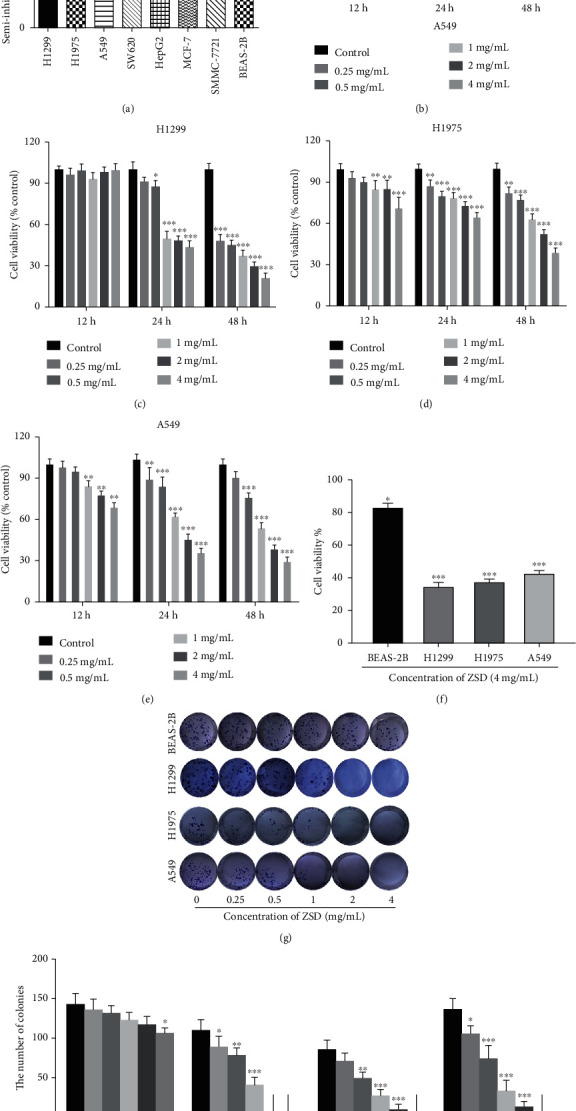
ZSD inhibited the proliferation of lung cancer cells *in vitro*. (a) Calculated IC50 of different cancer cells and normal human BEAS-2B cells. The cells were treated with various concentrations of ZSD (0.25, 0.5, 1, 2, and 4 mg/mL) for 48 h and IC50 was calculated. (b–e) ZSD inhibited the viability of BEAS-2B, H1299, H1975, A549 cells in a time and dose-dependent manner. BEAS-2B and lung cancer cells were treated with different concentrations of ZSD for 12 h, 24 h, and 48 h. The percentages of viable cells were determined using MTT assay. ^∗^*p* < 0.05, ^∗∗^*p* < 0.01, and ^∗∗∗^*p* < 0.001 vs. the control group. (f) 4 mg/mL of ZSD exhibited more prominent inhibitory effect on lung cancer cells than BEAS-2B cells. (g) ZSD reduced colony formation of lung cancer cells. Cells were treated with indicated concentrations of ZSD and cultured for 14 days to form colonies. Colonies were stained with crystal violet and then counted. (h) Quantitation analysis of colony formation numbers. ^∗^*p* < 0.05, ^∗∗^*p* < 0.01, and ^∗∗∗^*p* < 0.001 vs. the control group.

To further investigate the antiproliferative effect of ZSD on cancer cells, MTT assay was performed on cell lines from four types of cancer with high mortality or morbidity, including hepatocellular carcinoma (HepG2, SMMC-7721), breast cancer (MCF-7), colorectal cancer (SW620), and lung cancer (H1299, A549, H1975), and along with human normal lung epithelial cell BEAS-2B. In response to various concentrations of ZSD for 48 h, ZSD displayed a preferential inhibitory effect on the proliferation of lung cancer cells (H1299, H1975, and A549) with lower semi-inhibitory concentrations (IC50). However, the IC50 of BEAS-2B was much higher than that of lung cancer cell lines (Figure 3(a)), indicating that ZSD might exhibit specific inhibitory effect on lung cancer cells, so the dose-dependent viability studies were further performed. As shown in Figures 3(b)–3(e), ZSD inhibited the growth of H1299, H1975, and A549 cells in a time- and dose-dependent manner (*p* < 0.05; *p* < 0.01; *p* < 0.001), but had little toxicity on BEAS-2B cells (*p* < 0.05). At the concentration of 4 mg/mL, ZSD showed slight inhibitory effect on the proliferation of BEAS-2B cells, which was far less than that of lung cancer cells (Figure 3(f)). In addition, we also compared the inhibitory effect of ZSD with its single component (salvia and glycyrrhiza) on H1299 cells and found that the proliferation inhibition ratio of salvia and glycyrrhiza on H1299 was lower than that of ZSD at the same concentration (*p* < 0.01, *p* < 0.001; Figure S3), indicating that ZSD showed superior inhibitory effect on lung cancer cells to single component. Moreover, long-term cell viability assays (colony formation assays) showed that treatment with ZSD remarkably reduced the ability of lung cancer cells to form colonies, but had little effect on BEAS-2B cells (*p* < 0.01, *p* < 0.001; Figures 3(g) and 3(h)). These data showed that ZSD significantly decreased the viability of lung cancer cell lines in a time- and dose-dependent manner.

### 3.4. ZSD Inhibits the Migration and Invasion of Lung Cancer Cells

**Figure 4:**
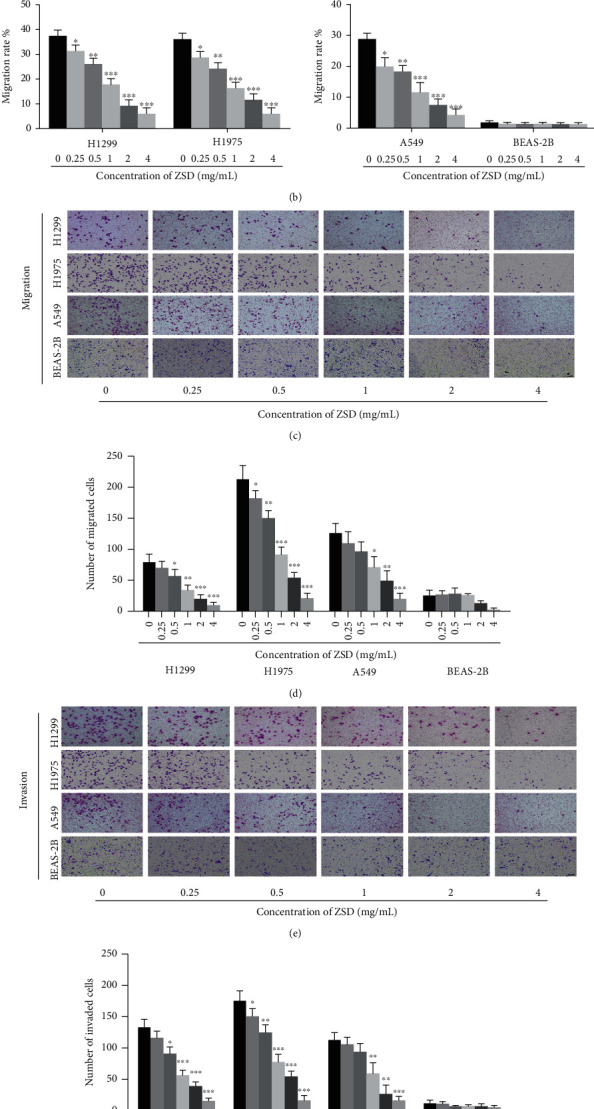
ZSD inhibited the migration and invasion of lung cancer cells. (a, b) ZSD inhibited mobility of lung cancer cells and had no effect on BEAS-2B cells in wound-healing assay. Scale bar: 200 *μ*m. (c, d) ZSD inhibited migration of lung cancer cells in a dose-dependent manner and showed no effect on BEAS-2B cells. Scale bar: 100 *μ*m. (e, f) ZSD inhibited invasion of lung cancer cells in a dose-dependent manner and showed no effect on BEAS-2B cells. Scale bar: 100 *μ*m. ^∗^*p* < 0.05, ^∗∗^*p* < 0.01, and ^∗∗∗^*p* < 0.001 vs. the control group.

Since metastasis constitutes the primary cause of poor clinical outcome, we wondered whether ZSD possessed anticancer function on tumor metastasis. The effect of ZSD on migration and invasion of H1299, A549, H1975 and BEAS-2B cells was evaluated by using wound-healing and transwell assays. As shown in Figures 4(a) and 4(b), the wound area was larger in the ZSD-treated group compared to the ZSD-free group, while it had no effect in BEAS-2B cells, suggesting that ZSD notably decreased the motility of H1299, A549, and H1975 cells in a dose-dependent manner (*p* < 0.05; *p* < 0.01; *p* < 0.001). Likewise, in the transwell assay, the numbers of migrating and invading cells were significantly reduced after treatment with ZSD in a concentration-dependent manner (*p* < 0.05, *p* < 0.01, and *p* < 0.001; Figures 4(c)–4(f)). Only a small number of BEAS-2B cells migrated or invaded to the lower chamber in transwell assay, and ZSD showed no effect on the migrating and invading BEAS-2B cells. These data demonstrated that ZSD might possess antimetastasis function in lung cancer cells.

### 3.5. ZSD Induces Apoptosis of H1299 Cells Partially by Regulating AKT/GSK-3*β*/*β*-Catenin Signaling

**Figure 5:**
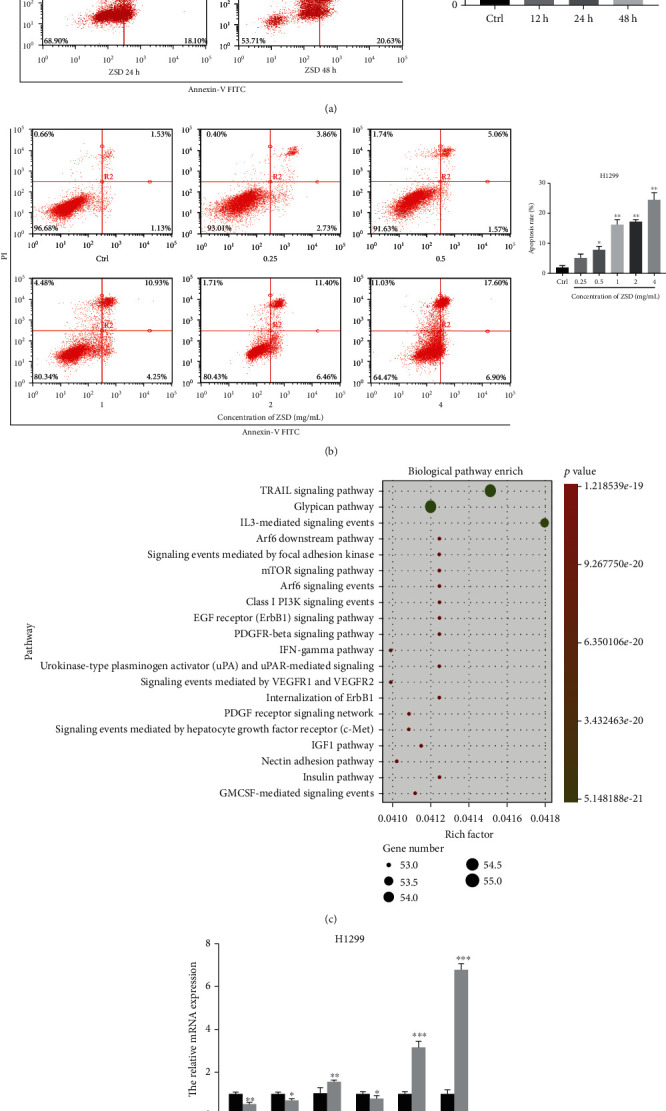
ZSD induced the apoptosis of H1299 cells partially by regulating AKT/GSK-3*β*/*β*-catenin signaling. (a) ZSD induced the apoptosis of H1299 cells after treatment with ZSD (4 mg/mL) for 12 h, 24 h, and 48 h. (b) ZSD induced apoptosis of H1299 cells in a dose-dependent manner. (c) Pathway enrichment analysis of the genes in Human Signal Transduction Pathway Finder PCR Array. (d) The mRNA expression of AKT/GSK-3*β*/*β*-catenin signal cascades in H1299 cells after treatment with ZSD (4 mg/mL). ^∗^*p* < 0.05 and ^∗∗^*p* < 0.01 vs. the control group.

To gain insight and determine the underlying regulatory mechanisms involved in the ZSD anticarcinogenic effect, we conducted Human Signal Transduction Pathway Finder RT^2^ Profiler™ PCR Array with RNA isolated from H1299 cells after ZSD treatment for 24 h. The PCR Array identified 4 upregulated genes (fold change ≥ 2) and 16 downregulated genes (fold change ≤ 0.5) in the ZSD treatment group (Table S4). Among them, antiapoptosis factor Bcl-2 was the most significant downregulated gene (fold change = 0.11). Next, we used flow cytometry to study deeply the apoptosis-inducing effect of ZSD. As shown in Figure 5(a), ZSD treatment (4 mg/mL) significantly increased the apoptosis rate of H1299 cells in a time-dependent manner (*p* < 0.05). Then, H1299 cells were treated with different concentrations of ZSD and the results showed that ZSD could induce apoptosis of H1299 cells in a dose-dependent manner (*p* < 0.05, *p* < 0.01; Figure 5(b)). Through PCR array detection, biological processes and pathways of the genes were analyzed. This information was introduced into FunRich for enrichment analysis and plotted by “ggplot2” package in R software (Figure 5(c)). Pathways were mainly enriched in TRAIL, glypican, IL-3, focal adhesion kinase, mTOR, PI3K/AKT, and IFN-*γ* signal events, which are closely related to the AKT/GSK-*β*/*β*-catenin pathway. Our results showed that ZSD treatment led to a dramatic decrease in the transcription of PI3K, AKT, and *β*-catenin (*p* < 0.05, *p* < 0.01; Figure 5(d)) and an increase in that of caspase-3, Bax, and GSK-3*β* (*p* < 0.01, *p* < 0.001). These data suggested that AKT/GSK-3*β*/*β*-catenin signaling was involved in ZSD-induced apoptosis of lung cancer cells.

### 3.6. ZSD Inhibits the AKT/GSK-3*β*/*β*-Catenin Pathway in Lung Cancer Cells and Tumor Tissues

**Figure 6:**
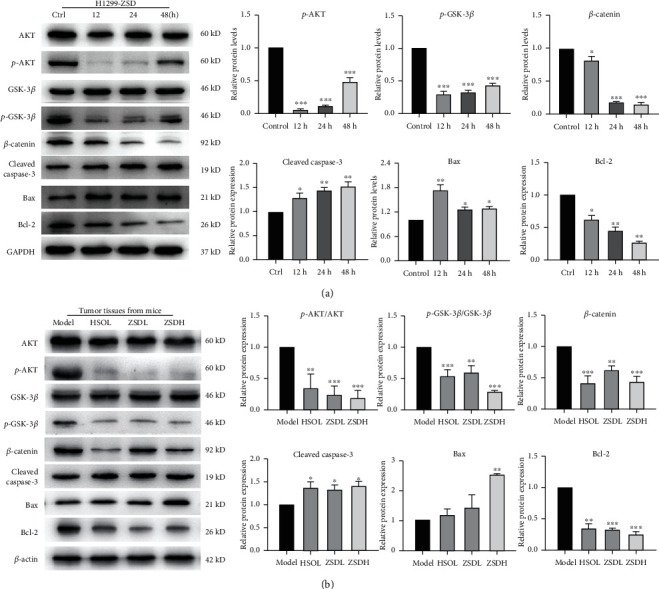
ZSD inhibited the AKT/GSK-3*β*/*β*-catenin pathway in lung cancer cells and tumor tissues. (a) Western blot analysis was used to assess the expression of multiple proteins involved in the AKT/GSK-3*β*/*β*-catenin pathway in lung cancer cells after ZSD treatment for 12 h, 24 h, and 48 h. ^∗^*p* < 0.05, ^∗∗^*p* < 0.01, and ^∗∗∗^*p* < 0.001 vs. the control group. (b) Western blots showing the protein expression of AKT, p-AKT, GSK-3*β*, p-GSK-3*β*, *β*-catenin, cleaved caspase-3, and Bax and Bcl-2 in isolated tumor tissues. ^∗^*p* < 0.05, ^∗∗^*p* < 0.01, and ^∗∗∗^*p* < 0.001 vs. the model group.

GSK-3*β*/*β*-catenin is considered to be an important downstream cascade of AKT in regulating cancer metastasis; thus, we investigated if ZSD inhibited growth and metastasis in lung cancer by modulating AKT/GSK-3*β*/*β*-catenin signaling. Our results showed that the protein level of p-AKT, p-GSK-3*β*, *β*-catenin, and Bcl-2 was markedly reduced in H1299 cells with ZSD treatment for 12 h, 24 h, and 48 h, while the level of cleaved caspase-3 and Bax was evidently increased compared to the control group (*p* < 0.05, *p* < 0.01, and *p* < 0.001; Figure 6(a)). Consistently, the samples from *in vivo* assay also showed that the protein expression of p-AKT, p-GSK-3*β*, *β*-catenin, and Bcl-2 significantly decreased (*p* < 0.01, *p* < 0.001), while the level of cleaved caspase-3 and Bax increased in the ZSD and HSOL treatment groups compared with the model group (*p* < 0.05, *p* < 0.01; Figure 6(b)). Furthermore, in the isolated allograft tumors, the mRNA expression level of PI3K, AKT, and *β*-catenin in the ZSD and HSOL treatment groups was downregulated compared with that in the model group, whereas that of GSK-3*β*, caspase-3, and Bax was upregulated (Figure S4). These findings indicated that ZSD may induce apoptosis and inhibit metastasis by blocking the AKT/GSK-3*β*/*β*-catenin pathway.

### 3.7. AKT/GSK-3*β*/*β*-Catenin Pathway Medicated the Anticancer Effect of ZSD in Lung Cancer Cells

**Figure 7:**
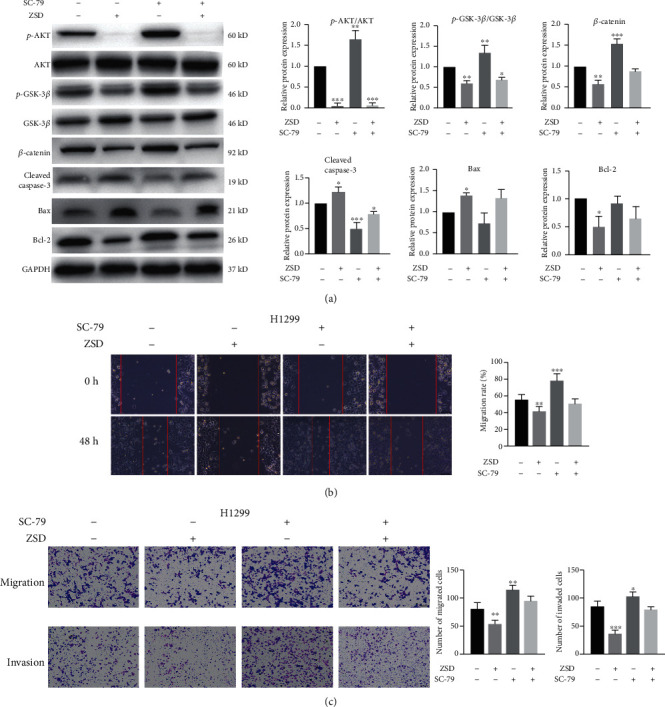
The AKT/GSK-3*β*/*β*-catenin pathway medicated anticancer effect of ZSD in lung cancer cells. H1299 cells were treated with ZSD and/or SC-79 for 24 h. (a) Representative western blots and quantitative analysis of the AKT/GSK-3*β*/*β*-catenin pathway related proteins. (b) The mobility of H1299 cells was detected by wound-healing assay. (c) The migration and invasion abilities of H1299 cells were detected by transwell assays. ^∗^*p* < 0.05, ^∗∗^*p* < 0.01, and ^∗∗∗^*p* < 0.001 vs. the control group.

To verify the role of AKT/GSK-3*β*/*β*-catenin in the anticancer effect of ZSD, we treated H1299 cells with ZSD and/or SC-79. SC-79 is a unique specific AKT activator, which effectively activates the phosphorylation of AKT [26]. Results in Figure 7(a) showed that SC-79 effectively promoted the protein expression of p-AKT and its downstream p-GSK-3*β* and *β*-catenin, which were significantly downregulated by ZSD. However, the pretreatment of SC-79 ameliorated the inhibition effect of ZSD on the expression of p-AKT, p-GSK-3*β*, Bcl-2, and *β*-catenin. In addition, the effect of ZSD on promoting the expression of apoptosis-related proteins (Bax, cleaved caspase-3) was reversed by pretreatment of SC-79 as well, compared with ZSD alone treatment. Furthermore, we also found that the migration rate of H1299 cells and migrated/invaded cell numbers in the ZSD and SC-79 cotreatment group were increased, compared with those in the ZSD alone treatment (Figures 7(b) and 7(c)), suggesting that activation of AKT weakened the ability of ZSD to inhibit metastasis. These results suggested that AKT/GSK-3*β*/*β*-catenin mediated anticancer effect of ZSD on lung cancer.

## 4. Discussion

Currently, although incremental progress has been made in the treatment of lung cancer, it remains a leading cause of human mortality. Surgical and chemotherapy treatment are the major treatment modalities for lung cancer patients. However, since chemotherapeutics have the insurmountable problems, such as drug resistance and systemic toxic effect, more effective therapeutic measures with little side effect are therefore required for the treatment of patients. The application of TCM is attracting more and more attention in the treatment of cancer for the advantages in improving quality of life, enhancing immune function, and reducing side effects of chemotherapy [27]. However, the TCM formula is a complicated system, and multicomponents and multitargets are the main characteristics. Therefore, elucidating the underlying therapeutic mechanisms is still a challenge. As network pharmacology provided an excellent method in collecting active ingredients and pharmacological actions of herbs or TCM formula [28], here, we integrated network pharmacology analysis and experimental validation to probe the effect of ZSD on lung cancer.

In this study, a total of 135 ingredients from ZSD were selected from the TCMSP database. Then, we identified 28 common targets from BATMAN-TCM, SEA, and SWISS databases. The GO function analysis showed that 28 potential targets are enriched for various biological processes, including cell response to steroid and transcription initiation from RNA polymerase II. Furthermore, KEGG pathway analysis found that ZSD may affect various cancers, including non-small-cell lung cancer pathway, acute myeloid leukemia pathway, and breast cancer. The three key targets in the NSCLC pathway were docked with the compounds, and the results showed that the compounds could be attached to the active pocket of the three key proteins. Next, we explored the effect of ZSD on lung cancer *in vitro* and *in vivo*.

The LLC subcutaneous transplantation model in C57BL/6 mice is a recognized classic lung cancer transplantation model with the advantages of high tumor formation rate and good repeatability [23]. Our results discovered for the first time that ZSD suppressed the tumor growth *in vivo* and high dose of ZSD exerted more prominent effect than HSOL treatment, which was used as an anticancer drug in clinics for years. Encouragingly, during the three-week period of ZSD and HSOL treatment, the mice showed no weight loss, decreased activity, or anorexia, implying ZSD can offer a safe treatment strategy for lung cancer. Compared with the model group, the ZSDL group showed no significant difference in the inhibitory effect, which is probably due to the large variance within the group or insufficient dose of ZSD.

Besides these observations, ZSD also played an active role in suppressing the proliferation and vitality, colony formation, migration, and invasion of lung cancer cells (A549, H1299, and H1975), but had no significant toxicity to normal lung epithelial cell (BEAS-2B). To further explore the antilung cancer mechanism of ZSD, we conducted Human Signal Transduction Pathway Finder PCR Array and found that TRAIL, glypican, IL-3-mediated signaling, Arf6 and mTOR signaling, and PI3K/AKT signaling pathway were mainly enriched (Figure 5(a)). Studies have shown that TRAIL and IL-3 signaling are involved in cell survival and proliferation via regulating the PI3K/AKT pathway [29–31]. Then, glypicans and Arf6 may promote tumor cell migration and metastasis through affecting the degradation of *β*-catenin in the Wnt pathway [32–34]. Thus, we paid more attention to the PI3K/AKT and Wnt/*β*-catenin pathways. AKT/GSK-3*β*/*β*-catenin is the “crosstalk” between the PI3K/AKT and Wnt/*β*-catenin pathways. It has been reported that activation of the GSK-3*β*/*β*-catenin pathway promotes the cell proliferation and leads to development of lung cancer [35, 36]. Our results showed that ZSD treatment significantly decreased the expression level of p-AKT, p-GSK-3*β*, and *β*-catenin and increased that of ROS-induced mitochondria-dependent apoptosis Bax/Bcl-2 and cleaved caspase-3 in lung cancer cells both *in vivo* and *in vitro*, indicating that ZSD treatment may induce cancer cell apoptosis via the AKT/GSK-3*β*/*β*-catenin pathway. Then, the activation of the AKT/GSK-3*β* pathway by SC-79 partially abolished the effect of ZSD on inducing apoptosis and on suppressing cell migration and invasion in lung cancer cells. These phenomena confirmed the critical role of the AKT/GSK-3*β*/*β*-catenin pathway in ZSD against lung cancer.

In the C-T network and KEGG pathway analysis, the targets enriched in the NSCLC pathway were AKT1, EGFR, and ALK. The molecular docking results revealed that bioactive components from ZSD have good affinity with these targets. Both EGFR and ALK can directly activate the PI3K/AKT pathway and the downstream effector GSK-3*β*/*β*-catenin [37–39]. The targets predicted by bioinformatics were consistent with our experiment results. Hence, our findings also provided a basis for mining the specific antitumor components of ZSD against lung cancer in follow-up work.

## 5. Conclusions

Taken together, our experimental results showed for the first time that ZSD exerted specific anticancer activity in lung cancer cells partially through inhibition of the AKT/GSK-3*β*/*β*-catenin signaling pathway *in vitro* and *in vivo*. These findings provide new evidence that ZSD has therapeutic potential in the treatment of lung cancer and uncover the potential mechanism by which ZSD inhibits human lung cancer cell growth.

## Data Availability

The data used to support the findings of this study are available from the corresponding author upon request.

## Supplementary Materials

Supplementary Materials Fig. S1 HPLC analysis of ZSD aqueous extract. Fig. S2 The thymus index of mice and the concentration of ALT and AST in each group. Fig. S3 The effect of salvia and glycyrrhiza on the viability of H1299 cells. Fig. S4 ZSD inhibited the AKT/GSK-3*β*/*β*-catenin pathway in LLC-allograft tumors. Table S1 Primer list of genes for RT-PCR in vitro and in vivo. Table S2 The gene ontology (GO) function enrichment analysis of potential targets. Table S3 KEGG pathway enrichment analyses of potential targets. Table S4 Up-and downregulated genes in the ZSD group compared with the control group.
