# Supplementary material for: Zi Shen Decoction Inhibits Growth and Metastasis of Lung Cancer via Regulating the AKT/GSK-3β/β-Catenin Pathway
Source: Oxid Med Cell Longev. 2021 Mar 9;2021:6685282. doi: 10.1155/2021/6685282 (PMC7969097; doi:10.1155/2021/6685282)
Supplement: Supplementary Materials — Fig. S1 HPLC analysis of ZSD aqueous extract. Fig. S2 The thymus index of mice and the concentration of ALT and AST in each group. Fig. S3 The effect of salvia and glycyrrhiza on the viability of H1299 cells. Fig. S4 ZSD inhibited the AKT/GSK-3β/β-catenin pathway in LLC-allograft tumors. Table S1 Primer list of genes for RT-PCR in vitro and in vivo. Table S2 The gene ontology (GO) function enrichment analysis of potential targets. Table S3 KEGG pathway enrichment analyses of potential targets. Table S4 Up-and downregulated genes in the ZSD group compared with the control group. [file 6685282.f1.pdf]

Zi Shen Decoction inhibits growth and metastasis of lung cancer via regulating AKT/GSK-3β/β-catenin pathway

a

b

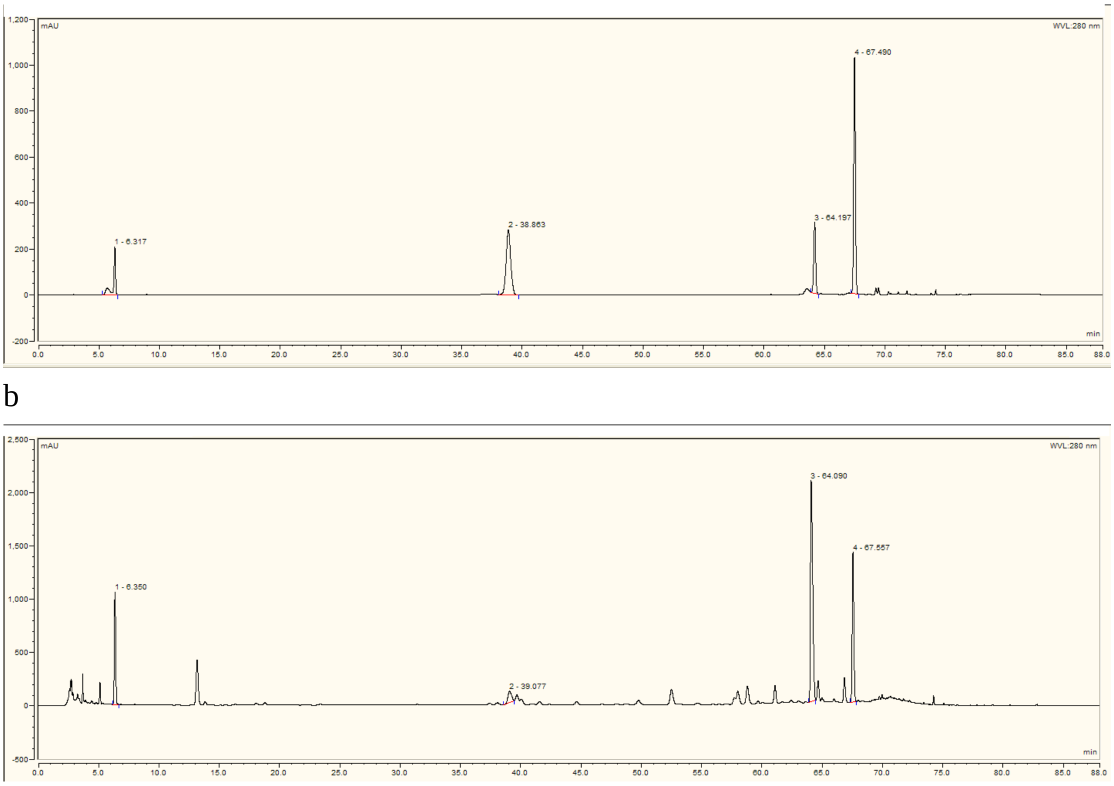

Fig. S1 HPLC analysis of ZSD aqueous extract. (a) HPLC of reference compound. (b) HPLC of ZSD aqueous extract.

a

b

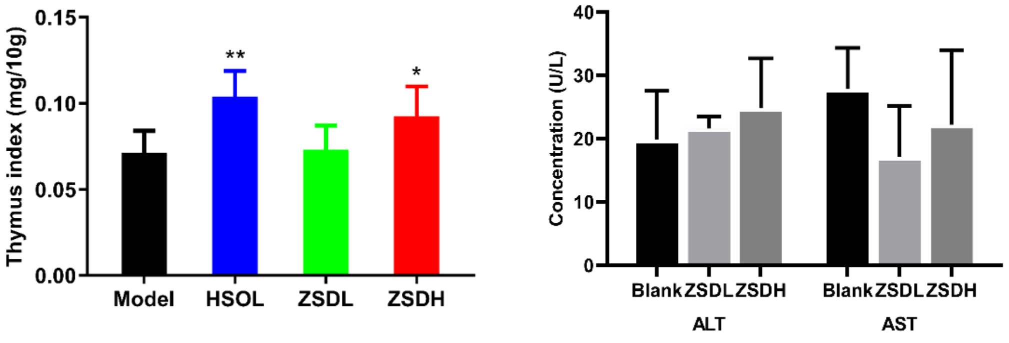

Fig. S2 The effect of ZSD on the thymus index and concentrations of ALT and AST of mice in each group. (a) The thymus index of each mouse was calculated and the results showed that the average index were significantly increased in ZSDH and HSOL treatment group compared with model group. (b) The concentrations of ALT and AST in blank mice and ZSD treatment mice. * $p < 0.05$, ** $p < 0.01$ vs. model group.

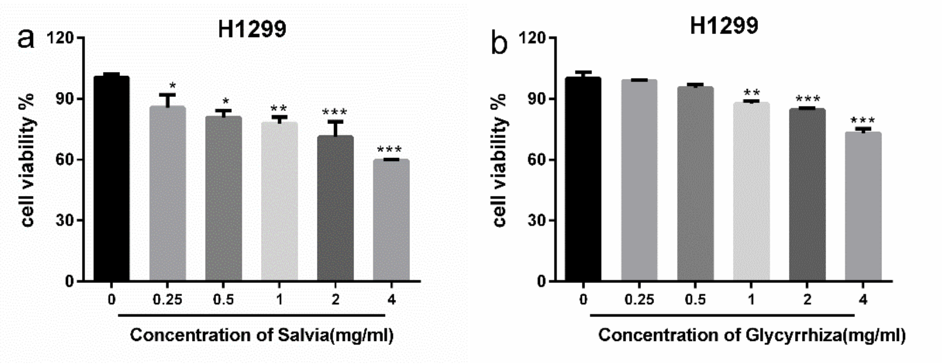

Fig. S3 The effect of salvia and glycyrrhiza on the viability of H1299 cells. Cell viability was analyzed by MTT assay. (a) The cell viability of H1299 cells was decreased with increasing concentrations of salvia for 48 h. (b) The cell viability of H1299 cells was decreased with increasing concentrations of glycyrrhiza for 48h. * $p < 0.05$, ** $p < 0.01$, *** $p < 0.001$ vs. control group.

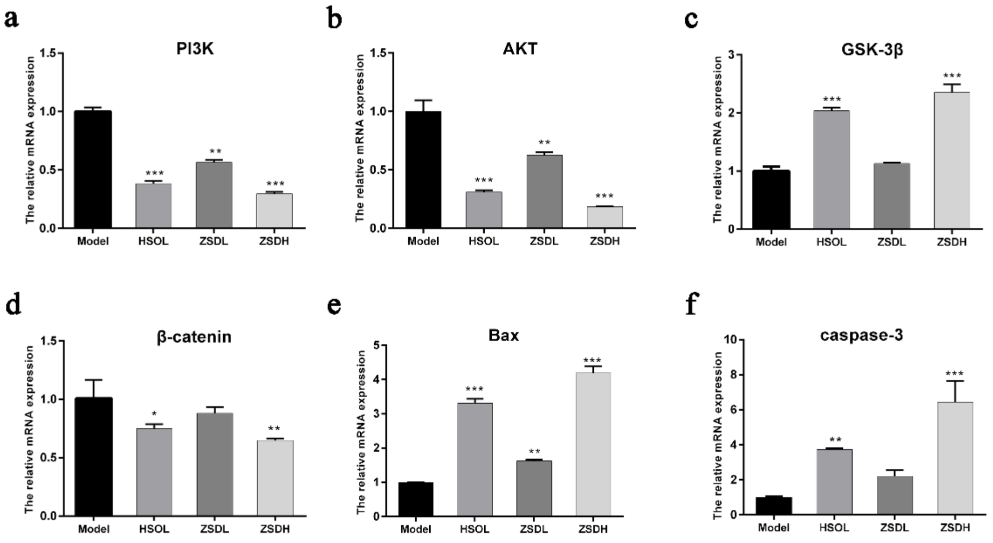

Fig. S4 ZSD blocked AKT/GSK-3β/β-catenin pathway in LLC-allograft tumors. The mRNA expression of PI3K, AKT, GSK-3β, β-catenin, caspase3, Bax was determined by RT-PCR. (a) The mRNA expression of PI3K was significantly down-regulated in HSOL and ZSDH treatment group. (b) The mRNA expression of AKT was significantly down-regulated in HSOL and ZSDH treatment group. (c) The mRNA expression of GSK-3β was significantly up-regulated in HSOL and ZSDH treatment group. (d) The mRNA expression of β-catenin was significantly down-regulated in

HSOL and ZSDH treatment group. (e) The mRNA expression of Bax was significantly up-regulated in HSOL and ZSDH treatment group. (f) The mRNA expression of caspase-3 was significantly up-regulated in HSOL and ZSDH treatment group. * $p < 0.05$, ** $p < 0.01$, *** $p < 0.001$ vs. model group.

Table S1 Primer list of genes for RT-PCR in vitro and vivo

mRNA	Primer sequence	species
PI3K	Sense (5'-3'): ACTTCACGGCATTCAGTTTGA<br>Anti-sense (5'-3'): GAGCACTTGGTAATCGGAGGA	Homo sapiens
AKT	Sense (5'-3'): TGAGCGACGTGGCTATTG<br>Anti-sense (5'-3'): CAGTCTGGATGGCGGTT	Homo sapiens
GSK-3β	Sense (5'-3'): GAGGAGGAATAAGGATGGTAGCC<br>Anti-sense (5'-3'): ACGGGACCCAAATGTCAAAC	Homo sapiens
β-catenin	Sense (5'-3'): CAGTGGGATGGTGGGTGTAAG<br>Anti-sense (5'-3'): CAGGAAGGGATGGAAGGTCTC	Homo sapiens
Bax	Sense (5'-3'): TGGAAGAAGATGGGCTGAGGC<br>Anti-sense (5'-3'): CATTCCCACCCCTCCCAATAAT	Homo sapiens
β-actin	Sense (5'-3'): CCCATGCCATCCTCCGTCTG<br>Anti-sense (5'-3'): TCTCGGCTGTGGTGGTGAAG	Homo sapiens
PI3K	Anti-sense (5'-3'): TTCCCTCGCAATAGGTTCTCC<br>Sense (5'-3'): GACCAATACTTGATGTGGCTGAC	mus musculus
AKT	Anti-sense (5'-3'): TCATCGAAATACCTGGTGTCAGTC<br>Sense (5'-3'): ACGGGCACATCAAGATAACGG	mus musculus
GSK-3β	Anti-sense (5'-3'): TTATTGGTCTGTCCACGGTCT<br>Sense (5'-3'): ACCATCCTTATCCCTCCACA	mus musculus
β-catenin	Sense (5'-3'): GGGCAACCCTGAGGAAGAAGA<br>Anti-sense (5'-3'): TGGGATGAGCAGCGTCAAACT	mus musculus
Caspase-3	Sense (5'-3'): CTGACTGGAAAGCCGAAACTC<br>Anti-sense (5'-3'):AGGGACTGGATGAACCACGAC	mus musculus
Bax	Sense (5'-3'): AATGCCCGTTCATCTCAG<br>Anti-sense (5'-3'): GGGACATCAGTCGCTTC	mus musculus
β-actin	Sense (5'-3'): GGTGTGATGGTGGGAATGGG<br>Anti-sense (5'-3'): ACGGTTGGCCTTAGGGTTCAG	mus musculus

Table S2 The gene ontology (GO) function enrichment analysis of potential targets

ONTOLOGY	ID	Description	GeneRatio	pvalue	p.adjust	qvalue	Count
BP	GO:0071383	cellular response to steroid hormone	11/27	2.15E-14	3.78E-11	1.69E-11	11
BP	GO:0043401	steroid hormone mediated signaling	10/27	4.93E-14	3.78E-11	1.69E-11	10
BP	GO:0006367	transcription initiation from RNA polymerase II promoter	10/27	6.13E-14	3.78E-11	1.69E-11	10
BP	GO:0030522	intracellular receptor signaling	11/27	7.48E-14	3.78E-11	1.69E-11	11
BP	GO:0009755	hormone-mediated signaling pathway	10/27	5.76E-13	2.33E-10	1.04E-10	10
BP	GO:0006352	DNA-templated transcription,	10/27	1.03E-12	3.46E-10	1.54E-10	10
BP	GO:0048545	response to steroid hormone	11/27	2.41E-12	6.97E-10	3.11E-10	11
BP	GO:0071222	cellular response to	8/27	3.42E-10	8.65E-08	3.86E-08	8
BP	GO:0071219	cellular response to molecule of	8/27	4.47E-10	1.00E-07	4.48E-08	8
BP	GO:0032496	response to lipopolysaccharide	9/27	5.35E-10	1.08E-07	4.83E-08	9
BP	GO:0050727	regulation of inflammatory response	10/27	7.24E-10	1.27E-07	5.65E-08	10
BP	GO:0002237	response to molecule of bacterial	9/27	7.52E-10	1.27E-07	5.65E-08	9
BP	GO:0071216	cellular response to biotic stimulus	8/27	1.04E-09	1.63E-07	7.25E-08	8
BP	GO:0015718	monocarboxylic acid transport	7/27	2.50E-09	3.60E-07	1.61E-07	7
BP	GO:0015908	fatty acid transport	6/27	4.56E-09	6.14E-07	2.74E-07	6
BP	GO:0010885	regulation of cholesterol storage	4/27	6.24E-09	7.88E-07	3.52E-07	4
BP	GO:0010878	cholesterol storage	4/27	1.05E-08	1.24E-06	5.55E-07	4
BP	GO:0010888	negative regulation of lipid storage	4/27	1.32E-08	1.49E-06	6.64E-07	4
BP	GO:0051235	maintenance of location	8/27	1.45E-08	1.50E-06	6.69E-07	8
BP	GO:0015849	organic acid transport	8/27	1.56E-08	1.50E-06	6.69E-07	8
BP	GO:0046942	carboxylic acid transport	8/27	1.56E-08	1.50E-06	6.69E-07	8
BP	GO:0031667	response to nutrient levels	9/27	1.98E-08	1.78E-06	7.92E-07	9
BP	GO:0007584	response to nutrient	8727	2.02E-08	1.78E-06	7.92E-07	7
BP	GO:1905953	negative regulation of lipid	5/27	2.41E-08	2.03E-06	9.04E-07	5
BP	GO:0006869	lipid transport	8/27	3.17E-08	2.47E-06	1.10E-06	8
BP	GO:0032102	negative regulation of response to external stimulus	8/27	3.17E-08	2.47E-06	1.10E-06	8
BP	GO:0006631	fatty acid metabolic process	8/27	4.60E-08	3.45E-06	1.54E-06	8
BP	GO:0010876	lipid localization	8/27	6.43E-08	4.65E-06	2.07E-06	8
BP	GO:1905952	regulation of lipid localization	6/27	8.22E-08	5.73E-06	2.56E-06	6
BP	GO:0051090	regulation of DNA-binding transcription factor activity	8/27	1.16E-07	7.84E-06	3.50E-06	8
BP	GO:0001975	response to amphetamine	4/27	1.21E-07	7.91E-06	3.53E-06	4
BP	GO:0050728	negative regulation of inflammatory response	6/27	1.27E-07	8.04E-06	3.59E-06	6
BP	GO:0009410	response to xenobiotic stimulus	7/27	1.45E-07	8.87E-06	3.96E-06	7
BP	GO:0033273	response to vitamin	5/27	2.04E-07	1.21E-05	5.40E-06	5
BP	GO:0015711	organic anion transport	8/27	2.68E-07	1.55E-05	6.92E-06	8
BP	GO:0035265	organ growth	6/27	3.87E-07	2.17E-05	9.69E-06	6
BP	GO:0071229	cellular response to acid chemical	6/27	4.46E-07	2.41E-05	1.07E-05	6
BP	GO:0007212	dopamine receptor signaling pathway	4/27	4.53E-07	2.41E-05	1.07E-05	4
BP	GO:0043279	response to alkaloid	5/27	5.16E-07	2.68E-05	1.19E-05	5
BP	GO:0010883	regulation of lipid storage	4/27	5.43E-07	2.68E-05	1.19E-05	4
BP	GO:0014075	response to amine	4/27	5.43E-07	2.68E-05	1.19E-05	4
BP	GO:0010745	negative regulation of macrophage derived foam cell differentiation	3/27	7.64E-07	3.68E-05	1.64E-05	3
BP	GO:0097366	response to bronchodilator	4/27	8.27E-07	3.89E-05	1.74E-05	4
BP	GO:0032368	regulation of lipid transport	5/27	9.63E-07	4.29E-05	1.92E-05	5
BP	GO:0031348	negative regulation of defense	6/27	9.77E-07	4.29E-05	1.92E-05	6
BP	GO:0033002	muscle cell proliferation	6/27	9.77E-07	4.29E-05	1.92E-05	6
BP	GO:0035296	regulation of tube diameter	5/27	1.73E-06	7.15E-05	3.19E-05	5
BP	GO:0050880	regulation of blood vessel size	5/27	1.73E-06	7.15E-05	3.19E-05	5
BP	GO:0097746	regulation of blood vessel diameter	5/27	1.73E-06	7.15E-05	3.19E-05	5
BP	GO:0035150	regulation of tube size	5/27	1.79E-06	7.25E-05	3.23E-05	5
BP	GO:0045834	positive regulation of lipid metabolic process	5/27	1.92E-06	7.61E-05	3.39E-05	5
BP	GO:0048608	reproductive structure development	7/27	1.98E-06	7.71E-05	3.44E-05	7
BP	GO:0061458	reproductive system development	7/27	2.08E-06	7.92E-05	3.53E-05	7
BP	GO:0062013	positive regulation of small molecule metabolic process	5/27	2.42E-06	9.05E-05	4.04E-05	5

BP	GO:0033189	response to vitamin A	3/27	2.57E-06	9.46E-05	4.22E-05	3
BP	GO:0019915	lipid storage	4/27	2.65E-06	9.57E-05	4.27E-05	4
BP	GO:0015909	long-chain fatty acid transport	4/27	2.81E-06	9.97E-05	4.45E-05	4
BP	GO:0048511	rhythmic process	6/27	3.31E-06	0.0001154	5.15E-05	6
BP	GO:0031670	cellular response to nutrient	4/27	3.72E-06	0.0001276	5.69E-05	4
BP	GO:0048660	regulation of smooth muscle cell proliferation	5/27	3.94E-06	0.0001326	5.92E-05	5
BP	GO:0010869	regulation of receptor biosynthetic process	3/27	4.08E-06	0.0001352	6.03E-05	3
BP	GO:0048659	smooth muscle cell proliferation	5/27	4.17E-06	0.0001359	6.06E-05	5
BP	GO:0016049	cell growth	7/27	4.27E-06	0.0001369	6.11E-05	7
BP	GO:0003018	vascular process in circulatory system	5/27	4.41E-06	0.0001394	6.22E-05	5
BP	GO:0007617	mating behavior	3/27	4.69E-06	0.0001458	6.50E-05	3
BP	GO:0010565	regulation of cellular ketone metabolic process	5/27	5.50E-06	0.0001686	7.52E-05	5
BP	GO:0008217	regulation of blood pressure	5/27	5.65E-06	0.0001706	7.61E-05	5
BP	GO:0048145	regulation of fibroblast proliferation	4/27	5.89E-06	0.0001753	7.82E-05	4
BP	GO:0048144	fibroblast proliferation	4/27	6.18E-06	0.0001812	8.08E-05	4
BP	GO:0032800	receptor biosynthetic process	3/27	6.86E-06	0.0001981	8.84E-05	3
BP	GO:0071496	cellular response to external stimulus	6/27	7.35E-06	0.0002094	9.34E-05	6
BP	GO:0001101	response to acid chemical	6/27	7.86E-06	0.0002178	9.72E-05	6
BP	GO:0072330	monocarboxylic acid biosynthetic process	6/27	7.86E-06	0.0002178	9.72E-05	6
BP	GO:0019217	regulation of fatty acid metabolic process	4/27	1.05E-05	0.0002839	0.0001266	4
BP	GO:0010743	regulation of macrophage derived foam cell differentiation	3/27	1.07E-05	0.0002839	0.0001266	3
BP	GO:0046320	regulation of fatty acid oxidation	3/27	1.07E-05	0.0002839	0.0001266	3
BP	GO:0007623	circadian rhythm	5/27	1.08E-05	0.000284	0.0001267	5
BP	GO:0001963	synaptic transmission, dopaminergic	3/27	1.18E-05	0.0003004	0.000134	3
BP	GO:0019098	reproductive behavior	3/27	1.18E-05	0.0003004	0.000134	3
BP	GO:0019395	fatty acid oxidation	4/27	1.19E-05	0.0003004	0.000134	4
BP	GO:0034440	lipid oxidation	4/27	1.29E-05	0.0003212	0.0001433	4
BP	GO:2000379	positive regulation of reactive oxygen species metabolic process	4/27	1.34E-05	0.0003299	0.0001472	4
BP	GO:0071868	cellular response to monoamine stimulus	3/27	1.57E-05	0.0003771	0.0001682	3
BP	GO:0071870	cellular response to catecholamine stimulus	3/27	1.57E-05	0.0003771	0.0001682	3
BP	GO:0051341	regulation of oxidoreductase activity	4/27	1.62E-05	0.0003846	0.0001716	4
BP	GO:0010742	macrophage derived foam cell differentiation	3/27	1.87E-05	0.0004336	0.0001934	3
BP	GO:0090077	foam cell differentiation	3/27	1.87E-05	0.0004336	0.0001934	3
BP	GO:0046777	protein autophosphorylation	5/27	1.95E-05	0.000448	0.0001999	5
BP	GO:0045923	positive regulation of fatty acid metabolic process	3/27	2.03E-05	0.0004608	0.0002056	3
BP	GO:0042752	regulation of circadian rhythm	4/27	2.08E-05	0.0004664	0.0002081	4
BP	GO:0019216	regulation of lipid metabolic process	6/27	2.16E-05	0.0004783	0.0002134	6
BP	GO:0071867	response to monoamine	3/27	2.20E-05	0.0004783	0.0002134	3
BP	GO:0071869	response to catecholamine	3/27	2.20E-05	0.0004783	0.0002134	3
BP	GO:0001558	regulation of cell growth	6/27	2.35E-05	0.0005053	0.0002254	6
BP	GO:0042180	cellular ketone metabolic process	5/27	2.52E-05	0.0005374	0.0002398	5
BP	GO:0007618	mating	3/27	2.57E-05	0.0005416	0.0002416	3
BP	GO:0007187	G protein-coupled receptor signaling pathway, coupled to cyclic nucleotide second messenger	5/27	2.94E-05	0.0006091	0.0002717	5
BP	GO:0048732	gland development	6/27	2.98E-05	0.0006091	0.0002717	6
BP	GO:0031641	regulation of myelination	3/27	2.98E-05	0.0006091	0.0002717	3
BP	GO:0050804	modulation of chemical synaptic transmission	6/27	3.06E-05	0.0006184	0.0002759	6
BP	GO:0099177	regulation of trans-synaptic signaling	6/27	3.10E-05	0.0006202	0.0002767	6
BP	GO:0051091	positive regulation of DNA-binding transcription factor activity	5/27	3.22E-05	0.0006393	0.0002852	5
BP	GO:0015850	organic hydroxy compound transport	5/27	3.28E-05	0.0006448	0.0002877	5
BP	GO:0007272	ensheathment of neurons	4/27	3.92E-05	0.0007493	0.0003343	4

BP	GO:0008366	axon ensheathment	4/27	3.92E-05	0.0007493	0.0003343	4
BP	GO:0045776	negative regulation of blood pressure	3/27	3.93E-05	0.0007493	0.0003343	3
BP	GO:0006874	cellular calcium ion homeostasis	6/27	4.03E-05	0.0007613	0.0003397	6
BP	GO:0061614	pri-miRNA transcription by RNA polymerase II	3/27	4.19E-05	0.0007798	0.0003479	3
BP	GO:0046394	carboxylic acid biosynthetic process	6/27	4.23E-05	0.0007798	0.0003479	6
BP	GO:0002683	negative regulation of immune system process	6/27	4.28E-05	0.0007798	0.0003479	6
BP	GO:0016053	organic acid biosynthetic process	6/27	4.28E-05	0.0007798	0.0003479	6
BP	GO:0030850	prostate gland development	3/27	4.47E-05	0.0008065	0.0003598	3
BP	GO:0033135	regulation of peptidyl-serine phosphorylation	4/27	4.52E-05	0.0008088	0.0003609	4
BP	GO:0055074	calcium ion homeostasis	6/27	4.71E-05	0.0008354	0.0003727	6
BP	GO:0050708	regulation of protein secretion	6/27	4.77E-05	0.0008379	0.0003738	6
BP	GO:0072593	reactive oxygen species metabolic process	5/27	4.82E-05	0.0008409	0.0003752	5
BP	GO:0030534	adult behavior	4/27	5.19E-05	0.0008967	0.0004001	4
BP	GO:0048146	positive regulation of fibroblast proliferation	3/27	5.36E-05	0.0009112	0.0004065	3
BP	GO:0050999	regulation of nitric-oxide synthase activity	3/27	5.36E-05	0.0009112	0.0004065	3
BP	GO:1903522	regulation of blood circulation	5/27	5.97E-05	0.0010053	0.0004485	5
BP	GO:0072503	cellular divalent inorganic cation homeostasis	6/27	6.07E-05	0.0010145	0.0004526	6
BP	GO:0018105	peptidyl-serine phosphorylation	5/27	6.16E-05	0.0010208	0.0004554	5
BP	GO:0001890	placenta development	4/27	6.41E-05	0.001053	0.0004698	4
BP	GO:0002791	regulation of peptide secretion	6/27	6.56E-05	0.0010704	0.0004776	6
BP	GO:0042220	response to cocaine	3/27	6.73E-05	0.0010887	0.0004857	3
BP	GO:0071398	cellular response to fatty acid	3/27	7.49E-05	0.0012022	0.0005364	3
BP	GO:0031663	lipopolysaccharide-mediated signaling pathway	3/27	7.89E-05	0.0012566	0.0005606	3
BP	GO:0097755	positive regulation of blood vessel diameter	3/27	8.31E-05	0.0013123	0.0005855	3
BP	GO:0006633	fatty acid biosynthetic process	4/27	8.60E-05	0.0013485	0.0006016	4
BP	GO:0018209	peptidyl-serine modification	5/27	8.74E-05	0.0013597	0.0006066	5
BP	GO:0007191	adenylate cyclase-activating dopamine receptor signaling pathway	2/27	9.00E-05	0.001389	0.0006197	2
BP	GO:0021543	pallium development	4/27	0.000103	0.0015753	0.0007028	4
BP	GO:0071902	positive regulation of protein serine/threonine kinase activity	5/27	0.000104	0.0015753	0.0007028	5
BP	GO:0032768	regulation of monooxygenase activity	3/27	0.000106	0.0015753	0.0007028	3
BP	GO:0045600	positive regulation of fat cell differentiation	3/27	0.000106	0.0015753	0.0007028	3
BP	GO:0060135	maternal process involved in female pregnancy	3/27	0.000106	0.0015753	0.0007028	3
BP	GO:0051583	dopamine uptake involved in synaptic transmission	2/27	0.00011	0.0016101	0.0007183	2
BP	GO:0051934	catecholamine uptake involved in synaptic transmission	2/27	0.00011	0.0016101	0.0007183	2
BP	GO:0010889	regulation of sequestering of triglyceride	2/27	0.000132	0.0019152	0.0008545	2
BP	GO:0071300	cellular response to retinoic acid	3/27	0.000133	0.0019152	0.0008545	3
BP	GO:0030308	negative regulation of cell growth	4/27	0.00014	0.0020062	0.0008951	4
BP	GO:0006635	fatty acid beta-oxidation	3/27	0.000151	0.0021134	0.0009429	3
BP	GO:0050805	negative regulation of synaptic transmission	3/27	0.000151	0.0021134	0.0009429	3
BP	GO:0060193	positive regulation of lipase activity	3/27	0.000151	0.0021134	0.0009429	3
BP	GO:0032754	positive regulation of interleukin-5 production	2/27	0.000156	0.0021167	0.0009443	2
BP	GO:1902894	negative regulation of pri-miRNA transcription by RNA polymerase II	2/27	0.000156	0.0021167	0.0009443	2
BP	GO:0033555	multicellular organismal response to stress	3/27	0.000157	0.0021167	0.0009443	3
BP	GO:0043627	response to estrogen	3/27	0.000157	0.0021167	0.0009443	3
BP	GO:1903524	positive regulation of blood	3/27	0.000157	0.0021167	0.0009443	3

BP	GO:0007565	female pregnancy	4.27	0.000158	0.0021167	0.0009443	4
BP	GO:0043112	receptor metabolic process	4/27	0.000158	0.0021167	0.0009443	4
BP	GO:1901654	response to ketone	4/27	0.000161	0.0021451	0.000957	4
BP	GO:2000377	regulation of reactive oxygen species metabolic process	4/27	0.000168	0.0022172	0.0009892	4
BP	GO:0001960	negative regulation of cytokine-mediated signaling pathway	3/27	0.00017	0.002231	0.0009953	3
BP	GO:0042310	vasoconstriction	3/27	0.000177	0.0023055	0.0010286	3
BP	GO:0060541	respiratory system development	4/27	0.000178	0.0023057	0.0010287	4
BP	GO:0071391	cellular response to estrogen stimulus	2/27	0.000181	0.0023353	0.0010419	2
BP	GO:0072347	response to anesthetic	3/27	0.000191	0.0024428	0.0010898	3
BP	GO:0030730	sequestering of triglyceride	2/27	0.000209	0.0026158	0.001167	2
BP	GO:0046321	positive regulation of fatty acid oxidation	2/27	0.000209	0.0026158	0.001167	2
BP	GO:0090494	dopamine uptake	2/27	0.000209	0.0026158	0.001167	2
BP	GO:0002064	epithelial cell development	4/27	0.000211	0.0026158	0.001167	4
BP	GO:0021766	hippocampus development	3/27	0.000213	0.0026158	0.001167	3
BP	GO:0033238	regulation of cellular amine metabolic process	3/27	0.000213	0.0026158	0.001167	3
BP	GO:0060761	negative regulation of response to cytokine stimulus	3/27	0.000213	0.0026158	0.001167	3
BP	GO:0046889	positive regulation of lipid biosynthetic process	3/27	0.000238	0.0028223	0.0012592	3
BP	GO:0097756	negative regulation of blood vessel diameter	3/27	0.000238	0.0028223	0.0012592	3
BP	GO:0032225	regulation of synaptic transmission, dopaminergic	2/27	0.000239	0.0028223	0.0012592	2
BP	GO:0060965	negative regulation of gene silencing by miRNA	2/27	0.000239	0.0028223	0.0012592	2
BP	GO:0071380	cellular response to prostaglandin E stimulus	2/27	0.000239	0.0028223	0.0012592	2
BP	GO:0090493	catecholamine uptake	2/27	0.000239	0.0028223	0.0012592	2
BP	GO:0044242	cellular lipid catabolic process	4/27	0.000253	0.0029689	0.0013246	4
BP	GO:0070542	response to fatty acid	3/27	0.000255	0.0029782	0.0013287	3
BP	GO:0010001	glial cell differentiation	4/27	0.000257	0.0029867	0.0013325	4
BP	GO:0032966	negative regulation of collagen biosynthetic process	2/27	0.00027	0.0031107	0.0013878	2
BP	GO:0007188	adenylate cyclase-modulating G protein-coupled receptor signaling	4/27	0.000271	0.0031107	0.0013878	4
BP	GO:0044706	multi-multicellular organism process	4/27	0.000275	0.0031467	0.0014039	4
BP	GO:0060333	interferon-gamma-mediated signaling pathway	3/27	0.000301	0.0034125	0.0015225	3
BP	GO:0010713	negative regulation of collagen metabolic process	2/27	0.000304	0.0034125	0.0015225	2
BP	GO:0031998	regulation of fatty acid beta-oxidation	2/27	0.000304	0.0034125	0.0015225	2
BP	GO:0003073	regulation of systemic arterial blood pressure	3/27	0.000321	0.0035654	0.0015907	3
BP	GO:1901655	cellular response to ketone	3/27	0.000321	0.0035654	0.0015907	3
BP	GO:0051047	positive regulation of secretion	5/27	0.000329	0.003637	0.0016226	5
BP	GO:0097305	response to alcohol	4/27	0.000331	0.0036381	0.0016231	4
BP	GO:0060149	negative regulation of posttranscriptional gene silencing	2/27	0.000339	0.0036484	0.0016277	2
BP	GO:0060438	trachea development	2/27	0.000339	0.0036484	0.0016277	2
BP	GO:0060967	negative regulation of gene silencing by RNA	2/27	0.000339	0.0036484	0.0016277	2
BP	GO:1903798	regulation of production of miRNAs involved in gene silencing by miRNA	2/27	0.000339	0.0036484	0.0016277	2
BP	GO:0042116	macrophage activation	3/27	0.000342	0.0036552	0.0016307	3
BP	GO:0042391	regulation of membrane potential	5/27	0.000351	0.0036998	0.0016507	5
BP	GO:0050673	epithelial cell proliferation	5/27	0.000351	0.0036998	0.0016507	5
BP	GO:0090277	positive regulation of peptide hormone secretion	3/27	0.000352	0.0036998	0.0016507	3
BP	GO:0031669	cellular response to nutrient levels	4/27	0.000353	0.0036998	0.0016507	4
BP	GO:0043434	response to peptide hormone	5/27	0.000358	0.0037208	0.00166	5
BP	GO:0030258	lipid modification	4/27	0.000359	0.0037208	0.00166	4

BP	GO:0060191	regulation of lipase activity	3/27	0.000374	0.0038071	0.0016985	3
BP	GO:0042053	regulation of dopamine metabolic process	2/27	0.000377	0.0038071	0.0016985	2
BP	GO:0042069	regulation of catecholamine metabolic process	2/27	0.000377	0.0038071	0.0016985	2
BP	GO:0048148	behavioral response to cocaine	2/27	0.000377	0.0038071	0.0016985	2
BP	GO:0051580	regulation of neurotransmitter uptake	2/27	0.000377	0.0038071	0.0016985	2
BP	GO:0007200	phospholipase C-activating G protein-coupled receptor signaling pathway	3/27	0.000397	0.0039959	0.0017827	3
BP	GO:0032649	regulation of interferon-gamma production	3/27	0.000409	0.0040737	0.0018175	3
BP	GO:0048661	positive regulation of smooth muscle cell proliferation	3/27	0.000409	0.0040737	0.0018175	3
BP	GO:0032674	regulation of interleukin-5 production	2/27	0.000416	0.0041016	0.0018299	2
BP	GO:0070920	regulation of production of small RNA involved in gene silencing by RNA	2/27	0.000416	0.0041016	0.0018299	2
BP	GO:0045088	regulation of innate immune response	5/27	0.000423	0.0041499	0.0018514	5
BP	GO:0045926	negative regulation of growth	4/27	0.000426	0.0041588	0.0018554	4
BP	GO:0021537	telencephalon development	4/27	0.000439	0.0042658	0.0019032	4
BP	GO:0019233	sensory perception of pain	3/27	0.000446	0.0043112	0.0019234	3
BP	GO:0062012	regulation of small molecule metabolic process	5/27	0.000454	0.0043505	0.0019409	5
BP	GO:0032634	interleukin-5 production	2/27	0.000457	0.0043505	0.0019409	2
BP	GO:0071379	cellular response to prostaglandin stimulus	2/27	0.000457	0.0043505	0.0019409	2
BP	GO:0033138	positive regulation of peptidyl-serine phosphorylation	3/27	0.000458	0.0043505	0.0019409	3
BP	GO:0007611	learning or memory	4/27	0.000473	0.0044666	0.0019927	4
BP	GO:0062014	negative regulation of small molecule metabolic process	3/27	0.000484	0.0045547	0.0020321	3
BP	GO:0043406	positive regulation of MAP kinase activity	4/27	0.000487	0.004557	0.0020331	4
BP	GO:0009062	fatty acid catabolic process	3/27	0.000498	0.004574	0.0020407	3
BP	GO:0032526	response to retinoic acid	3/27	0.000498	0.004574	0.0020407	3
BP	GO:0099565	chemical synaptic transmission, postsynaptic	3/27	0.000498	0.004574	0.0020407	3
BP	GO:1904659	glucose transmembrane transport	3/27	0.000498	0.004574	0.0020407	3
BP	GO:0034695	response to prostaglandin E	2/27	0.0005	0.0045755	0.0020413	2
BP	GO:0021761	limbic system development	3/27	0.000511	0.0046566	0.0020775	3
BP	GO:0006939	smooth muscle contraction	3/27	0.000525	0.0047398	0.0021146	3
BP	GO:0033559	unsaturated fatty acid metabolic process	3/27	0.000525	0.0047398	0.0021146	3
BP	GO:0008645	hexose transmembrane transport	3/27	0.000553	0.0049737	0.002219	3
BP	GO:0031668	cellular response to extracellular stimulus	4/27	0.000562	0.0049935	0.0022278	4
BP	GO:0050714	positive regulation of protein secretion	4/27	0.000562	0.0049935	0.0022278	4
BP	GO:0032609	interferon-gamma production	3/27	0.000568	0.0049935	0.0022278	3
BP	GO:0046620	regulation of organ growth	3/27	0.000568	0.0049935	0.0022278	3
BP	GO:0060964	regulation of gene silencing by	3/27	0.000568	0.0049935	0.0022278	3
BP	GO:0045927	positive regulation of growth	4/27	0.000578	0.005056	0.0022557	4
BP	GO:0006690	icosanoid metabolic process	3/27	0.000583	0.0050575	0.0022564	3
BP	GO:0015749	monosaccharide transmembrane transport	3/27	0.000583	0.0050575	0.0022564	3
BP	GO:0002068	glandular epithelial cell development	2/27	0.000592	0.0050664	0.0022604	2
BP	GO:0060330	regulation of response to interferon-gamma	2/27	0.000592	0.0050664	0.0022604	2
BP	GO:0060334	regulation of interferon-gamma-mediated signaling pathway	2/27	0.000592	0.0050664	0.0022604	2
BP	GO:0032868	response to insulin	4/27	0.000594	0.0050664	0.0022604	4
BP	GO:0061448	connective tissue development	4/27	0.000602	0.0051151	0.0022821	4
BP	GO:0021782	glial cell development	3/27	0.000613	0.0051654	0.0023045	3
BP	GO:0034219	carbohydrate transmembrane transport	3/27	0.000613	0.0051654	0.0023045	3
BP	GO:0043270	positive regulation of ion transport	4/27	0.000619	0.0051917	0.0023163	4
BP	GO:0060147	regulation of posttranscriptional gene silencing	3/27	0.000629	0.0052309	0.0023337	3

BP	GO:0060966	regulation of gene silencing by RNA	3/27	0.000629	0.0052309	0.0023337	3
BP	GO:0060740	prostate gland epithelium morphogenesis	2/27	0.000641	0.0053094	0.0023688	2
BP	GO:0050900	leukocyte migration	5/27	0.000663	0.0054733	0.0024419	5
BP	GO:0030100	regulation of endocytosis	4/27	0.000671	0.0055147	0.0024603	4
BP	GO:0022612	gland morphogenesis	3/27	0.000677	0.0055399	0.0024716	3
BP	GO:0098810	neurotransmitter reuptake	2/27	0.000691	0.0055915	0.0024946	2
BP	GO:1905208	negative regulation of cardiocyte differentiation	2/27	0.000691	0.0055915	0.0024946	2
BP	GO:2000191	regulation of fatty acid transport	2/27	0.000691	0.0055915	0.0024946	2
BP	GO:0002793	positive regulation of peptide	4/27	0.000736	0.0058798	0.0026232	4
BP	GO:0001516	prostaglandin biosynthetic process	2/27	0.000744	0.0058798	0.0026232	2
BP	GO:0046457	prostanoid biosynthetic process	2/27	0.000744	0.0058798	0.0026232	2
BP	GO:0060512	prostate gland morphogenesis	2/27	0.000744	0.0058798	0.0026232	2
BP	GO:0046717	acid secretion	3/27	0.000744	0.0058798	0.0026232	3
BP	GO:0051209	release of sequestered calcium ion into cytosol	3/27	0.000744	0.0058798	0.0026232	3
BP	GO:0042063	gliogenesis	4/27	0.000755	0.0059391	0.0026497	4
BP	GO:0051283	negative regulation of sequestering of calcium ion	3/27	0.00078	0.0061117	0.0027267	3
BP	GO:0045822	negative regulation of heart	2/27	0.000798	0.0062318	0.0027803	2
BP	GO:0050890	cognition	4/27	0.000815	0.0063239	0.0028214	4
BP	GO:0051282	regulation of sequestering of calcium ion	3/27	0.000816	0.0063239	0.0028214	3
BP	GO:0044106	cellular amine metabolic process	3/27	0.000835	0.0064237	0.0028659	3
BP	GO:0002758	innate immune response-activating signal transduction	4/27	0.000836	0.0064237	0.0028659	4
BP	GO:0048384	retinoic acid receptor signaling	2/27	0.000854	0.00652	0.0029089	2
BP	GO:0061037	negative regulation of cartilage development	2/27	0.000854	0.00652	0.0029089	2
BP	GO:0046887	positive regulation of hormone secretion	3/27	0.000873	0.0066113	0.0029496	3
BP	GO:0051208	sequestering of calcium ion	3/27	0.000873	0.0066113	0.0029496	3
BP	GO:0042552	myelination	3/27	0.000892	0.0066834	0.0029818	3
BP	GO:0045598	regulation of fat cell differentiation	3/27	0.000892	0.0066834	0.0029818	3
BP	GO:0072329	monocarboxylic acid catabolic process	3/27	0.000892	0.0066834	0.0029818	3
BP	GO:0034694	response to prostaglandin	2/27	0.000913	0.0067102	0.0029937	2
BP	GO:0045737	positive regulation of cyclin-dependent protein serine/threonine	2/27	0.000913	0.0067102	0.0029937	2
BP	GO:0050482	arachidonic acid secretion	2/27	0.000913	0.0067102	0.0029937	2
BP	GO:0060292	long-term synaptic depression	2/27	0.000913	0.0067102	0.0029937	2
BP	GO:1903963	arachidonate transport	2/27	0.000913	0.0067102	0.0029937	2
BP	GO:0032355	response to estradiol	3/27	0.000932	0.006829	0.0030467	3
BP	GO:0060359	response to ammonium ion	3/27	0.000952	0.0069523	0.0031018	3
BP	GO:0045907	positive regulation of vasoconstriction	2/27	0.000973	0.0069985	0.0031223	2
BP	GO:0051968	positive regulation of synaptic transmission, glutamatergic	2/27	0.000973	0.0069985	0.0031223	2
BP	GO:0055094	response to lipoprotein particle	2/27	0.000973	0.0069985	0.0031223	2
BP	GO:0071295	cellular response to vitamin	2/27	0.000973	0.0069985	0.0031223	2
BP	GO:0043010	camera-type eye development	4/27	0.001015	0.0072514	0.0032352	4
BP	GO:0009308	amine metabolic process	3/27	0.001015	0.0072514	0.0032352	3
BP	GO:0070588	calcium ion transmembrane transport	4/27	0.001027	0.0073113	0.0032619	4
BP	GO:0007189	adenylate cyclase-activating G protein-coupled receptor signaling	3/27	0.001036	0.0073522	0.0032802	3
BP	GO:0060078	regulation of postsynaptic membrane potential	3/27	0.001058	0.0074537	0.0033254	3
BP	GO:0097553	calcium ion transmembrane import into cytosol	3/27	0.001058	0.0074537	0.0033254	3
BP	GO:0002218	activation of innate immune response	4/27	0.001076	0.0075288	0.0033589	4
BP	GO:0007204	positive regulation of cytosolic calcium ion concentration	4/27	0.001076	0.0075288	0.0033589	4
BP	GO:0050709	negative regulation of protein	3/27	0.00108	0.0075297	0.0033593	3
BP	GO:0010765	positive regulation of sodium ion transport	2/27	0.001098	0.0076039	0.0033924	2

BP	GO:0071402	cellular response to lipoprotein particle stimulus	2/27	0.001098	0.0076039	0.0033924	2
BP	GO:0001893	maternal placenta development	2/27	0.001164	0.0079219	0.0035343	2
BP	GO:0032689	negative regulation of interferon-gamma production	2/27	0.001164	0.0079219	0.0035343	2
BP	GO:0034142	toll-like receptor 4 signaling pathway	2/27	0.001164	0.0079219	0.0035343	2
BP	GO:0051354	negative regulation of oxidoreductase activity	2/27	0.001164	0.0079219	0.0035343	2
BP	GO:1904031	positive regulation of cyclin-dependent protein kinase activity	2/27	0.001164	0.0079219	0.0035343	2
BP	GO:0002224	toll-like receptor signaling pathway	3/27	0.001194	0.0080749	0.0036026	3
BP	GO:0035264	multicellular organism growth	3/27	0.001194	0.0080749	0.0036026	3
BP	GO:0003417	growth plate cartilage development	3/27	0.001231	0.0082594	0.0036849	2
BP	GO:0071392	cellular response to estradiol stimulus	3/27	0.001231	0.0082594	0.0036849	2
BP	GO:0002792	negative regulation of peptide	3/27	0.001242	0.0082594	0.0036849	3
BP	GO:0008643	carbohydrate transport	3/27	0.001242	0.0082594	0.0036849	3
BP	GO:0014065	phosphatidylinositol 3-kinase	3/27	0.001242	0.0082594	0.0036849	3
BP	GO:0016042	lipid catabolic process	4/27	0.001261	0.0083342	0.0037182	4
BP	GO:0032147	activation of protein kinase activity	4/27	0.001261	0.0083342	0.0037182	4
BP	GO:0030336	negative regulation of cell migration	4/27	0.001275	0.0083989	0.0037471	4
BP	GO:0001662	behavioral fear response	2/27	0.0013	0.0083989	0.0037471	2
BP	GO:0030212	hyaluronan metabolic process	2/27	0.0013	0.0083989	0.0037471	2
BP	GO:0031076	embryonic camera-type eye development	2/27	0.0013	0.0083989	0.0037471	2
BP	GO:0038083	peptidyl-tyrosine autophosphorylation	2/27	0.0013	0.0083989	0.0037471	2
BP	GO:0045777	positive regulation of blood pressure	2/27	0.0013	0.0083989	0.0037471	2
BP	GO:0071276	cellular response to cadmium ion	2/27	0.0013	0.0083989	0.0037471	2
BP	GO:0055088	lipid homeostasis	3/27	0.001316	0.0084337	0.0037627	3
BP	GO:1903169	regulation of calcium ion transmembrane transport	3/27	0.001316	0.0084337	0.0037627	3
BP	GO:0043405	regulation of MAP kinase activity	4/27	0.001318	0.0084337	0.0037627	4
BP	GO:0002209	behavioral defense response	2/27	0.001371	0.0086639	0.0038653	2
BP	GO:0007190	activation of adenylate cyclase activity	2/27	0.001371	0.0086639	0.0038653	2
BP	GO:0032094	response to food	2/27	0.001371	0.0086639	0.0038653	2
BP	GO:1903523	negative regulation of blood	2/27	0.001371	0.0086639	0.0038653	2
BP	GO:0060968	regulation of gene silencing	3/27	0.001418	0.0089332	0.0039855	3
BP	GO:0042417	dopamine metabolic process	2/27	0.001444	0.0089837	0.004008	2
BP	GO:0055090	acylglycerol homeostasis	2/27	0.001444	0.0089837	0.004008	2
BP	GO:0060969	negative regulation of gene silencing	2/27	0.001444	0.0089837	0.004008	2
BP	GO:0070328	triglyceride homeostasis	2/27	0.001444	0.0089837	0.004008	2
BP	GO:0048638	regulation of developmental growth	4/27	0.001468	0.0091039	0.0040617	4
BP	GO:0070997	neuron death	4/27	0.001483	0.0091725	0.0040923	4
BP	GO:0060402	calcium ion transport into cytosol	3/27	0.001498	0.0092134	0.0041105	3
BP	GO:2000146	negative regulation of cell motility	4/27	0.001499	0.0092134	0.0041105	4
BP	GO:0042596	fear response	2/27	0.001519	0.0092768	0.0041388	2
BP	GO:0044058	regulation of digestive system process	2/27	0.001519	0.0092768	0.0041388	2
BP	GO:0032680	regulation of tumor necrosis factor production	3/27	0.001553	0.0094606	0.0042208	3
BP	GO:0001505	regulation of neurotransmitter levels	3/27	0.001579	0.0095906	0.0042788	4
BP	GO:1902893	regulation of pri-miRNA transcription by RNA polymerase II	2/27	0.001595	0.0096563	0.0043081	2
BP	GO:0051480	regulation of cytosolic calcium ion concentration	4/27	0.001629	0.0098294	0.0043853	4
BP	GO:0032640	tumor necrosis factor production	3/27	0.001638	0.0098294	0.0043853	3
BP	GO:1903555	regulation of tumor necrosis factor superfamily cytokine production	3/27	0.001638	0.0098294	0.0043853	3
BP	GO:0010907	positive regulation of glucose metabolic process	2/27	0.001673	0.0100102	0.004466	2
BP	GO:0001654	eye development	4/27	0.001714	0.0101438	0.0045256	4
BP	GO:0018108	peptidyl-tyrosine phosphorylation	4/27	0.001732	0.0101438	0.0045256	4
BP	GO:0006692	prostanoid metabolic process	2/27	0.001753	0.0101438	0.0045256	2
BP	GO:0006693	prostaglandin metabolic process	2/27	0.001753	0.0101438	0.0045256	2
BP	GO:0006775	fat-soluble vitamin metabolic process	2/27	0.001753	0.0101438	0.0045256	2
BP	GO:0010863	positive regulation of phospholipase C activity	2/27	0.001753	0.0101438	0.0045256	2

BP	GO:0045429	positive regulation of nitric oxide biosynthetic process	2/27	0.001753	0.0101438	0.0045256	2
BP	GO:0045746	negative regulation of Notch signaling pathway	2/27	0.001753	0.0101438	0.0045256	2
BP	GO:0060612	adipose tissue development	2/27	0.001753	0.0101438	0.0045256	2
BP	GO:0071364	cellular response to epidermal growth factor stimulus	2/27	0.001753	0.0101438	0.0045256	2
BP	GO:0035051	cardiocyte differentiation	3/27	0.001756	0.0101438	0.0045256	3
BP	GO:1901568	fatty acid derivative metabolic process	3/27	0.001756	0.0101438	0.0045256	3
BP	GO:0018212	peptidyl-tyrosine modification	4/27	0.001785	0.0102017	0.0045514	4
BP	GO:0150063	visual system development	4/27	0.001785	0.0102017	0.0045514	4
BP	GO:0050806	positive regulation of synaptic transmission	3/27	0.001786	0.0102017	0.0045514	3
BP	GO:0071706	tumor necrosis factor superfamily cytokine production	3/27	0.001786	0.0102017	0.0045514	3
BP	GO:0003416	endochondral bone growth	2/27	0.001835	0.0103553	0.00462	2
BP	GO:0014002	astrocyte development	2/27	0.001835	0.0103553	0.00462	2
BP	GO:0032965	regulation of collagen biosynthetic process	2/27	0.001835	0.0103553	0.00462	2
BP	GO:1904407	positive regulation of nitric oxide metabolic process	2/27	0.001835	0.0103553	0.00462	2
BP	GO:0006909	phagocytosis	4/27	0.001839	0.0103553	0.00462	4
BP	GO:0043433	negative regulation of DNA-binding transcription factor activity	3/27	0.001847	0.0103765	0.0046294	3
BP	GO:0048880	sensory system development	4/27	0.001875	0.0104933	0.0046815	4
BP	GO:0060401	cytosolic calcium ion transport	3/27	0.001879	0.0104933	0.0046815	3
BP	GO:0051098	regulation of binding	4/27	0.001912	0.0106302	0.0047426	4
BP	GO:0032309	icosanoid secretion	2/27	0.001919	0.0106302	0.0047426	2
BP	GO:1900274	regulation of phospholipase C activity	2/27	0.001919	0.0106302	0.0047426	2
BP	GO:0001659	temperature homeostasis	3/27	0.001942	0.0107286	0.0047865	3
BP	GO:0001504	neurotransmitter uptake	2/27	0.002004	0.0109116	0.0048682	2
BP	GO:0002067	glandular epithelial cell differentiation	2/27	0.002004	0.0109116	0.0048682	2
BP	GO:0032369	negative regulation of lipid transport	2/27	0.002004	0.0109116	0.0048682	2
BP	GO:0035196	production of miRNAs involved in gene silencing by miRNA	2/27	0.002004	0.0109116	0.0048682	2
BP	GO:0043277	apoptotic cell clearance	2/27	0.002004	0.0109116	0.0048682	2
BP	GO:0050678	regulation of epithelial cell	4/27	0.002007	0.0109116	0.0048682	4
BP	GO:0030900	forebrain development	4/27	0.002066	0.0111702	0.0049835	4
BP	GO:0045089	positive regulation of innate immune response	4/27	0.002066	0.0111702	0.0049835	4
BP	GO:0001959	regulation of cytokine-mediated signaling pathway	3/27	0.002073	0.0111758	0.004986	3
BP	GO:0060351	cartilage development involved in endochondral bone morphogenesis	2/27	0.002092	0.0111882	0.0049915	2
BP	GO:0070849	response to epidermal growth factor	2/27	0.002092	0.0111882	0.0049915	2
BP	GO:0098868	bone growth	2/27	0.002092	0.0111882	0.0049915	2
BP	GO:0051271	negative regulation of cellular component movement	4/27	0.002126	0.0113419	0.0050602	4
BP	GO:0071346	cellular response to interferon-gamma	3/27	0.002174	0.011569	0.0051615	3
BP	GO:0060324	face development	2/27	0.002181	0.0115724	0.005163	2
BP	GO:0048015	phosphatidylinositol-mediated	3/27	0.002209	0.0116912	0.005216	3
BP	GO:0045787	positive regulation of cell cycle	4/27	0.002228	0.0117638	0.0052484	4
BP	GO:0002686	negative regulation of leukocyte migration	2/27	0.002271	0.0117759	0.0052538	2
BP	GO:0003044	regulation of systemic arterial blood pressure mediated by a chemical	2/27	0.002271	0.0117759	0.0052538	2
BP	GO:0010712	regulation of collagen metabolic	2/27	0.002271	0.0117759	0.0052538	2
BP	GO:0043124	negative regulation of I-kappaB kinase/NF-kappaB signaling	2/27	0.002271	0.0117759	0.0052538	2
BP	GO:0071715	icosanoid transport	2/27	0.002271	0.0117759	0.0052538	2
BP	GO:1901571	fatty acid derivative transport	2/27	0.002271	0.0117759	0.0052538	2
BP	GO:1990090	cellular response to nerve growth factor stimulus	2/27	0.002271	0.0117759	0.0052538	2
BP	GO:0038061	NIK/NF-kappaB signaling	3/27	0.002279	0.0117844	0.0052575	3
BP	GO:0048017	inositol lipid-mediated signaling	3/27	0.002314	0.0119073	0.0053124	3

BP	GO:0048639	positive regulation of developmental growth	3/27	0.002314	0.0119073	0.0053124	3
BP	GO:0010959	regulation of metal ion transport	4/27	0.002334	0.0119475	0.0053303	4
BP	GO:0070482	response to oxygen levels	4/27	0.002334	0.0119475	0.0053303	4
BP	GO:1900087	positive regulation of G1/S transition of mitotic cell cycle	2/27	0.002364	0.01207	0.005385	2
BP	GO:0040013	negative regulation of locomotion	4/27	0.002377	0.0121076	0.0054017	4
BP	GO:0019933	cAMP-mediated signaling	3/27	0.002423	0.0122791	0.0054783	3
BP	GO:0048167	regulation of synaptic plasticity	3/27	0.002423	0.0122791	0.0054783	3
BP	GO:1903532	positive regulation of secretion by cell	4/27	0.002443	0.0123334	0.0055025	4
BP	GO:0019369	arachidonic acid metabolic process	2/27	0.002458	0.0123334	0.0055025	2
BP	GO:0031050	dsRNA processing	2/27	0.002458	0.0123334	0.0055025	2
BP	GO:0070918	production of small RNA involved in gene silencing by RNA	2/27	0.002458	0.0123334	0.0055025	2
BP	GO:0060759	regulation of response to cytokine stimulus	3/27	0.002535	0.0126869	0.0056602	3
BP	GO:0015872	dopamine transport	2/27	0.002554	0.0127206	0.0056753	2
BP	GO:1990089	response to nerve growth factor	2/27	0.002554	0.0127206	0.0056753	2
BP	GO:0007219	Notch signaling pathway	3/27	0.00265	0.0131109	0.0058493	3
BP	GO:0051224	negative regulation of protein	3/27	0.00265	0.0131109	0.0058493	3
BP	GO:0032964	collagen biosynthetic process	2/27	0.002652	0.0131109	0.0058493	2
BP	GO:0006584	catecholamine metabolic process	2/27	0.002752	0.0134387	0.0059956	2
BP	GO:0006636	unsaturated fatty acid biosynthetic process	2/27	0.002752	0.0134387	0.0059956	2
BP	GO:0009712	catechol-containing compound metabolic process	2/27	0.002752	0.0134387	0.0059956	2
BP	GO:0032663	regulation of interleukin-2 production	2/27	0.002752	0.0134387	0.0059956	2
BP	GO:0050994	regulation of lipid catabolic process	2/27	0.002752	0.0134387	0.0059956	2
BP	GO:0002221	pattern recognition receptor signaling pathway	3/27	0.002808	0.0136492	0.0060895	3
BP	GO:1904950	negative regulation of establishment of protein localization	3/27	0.002808	0.0136492	0.0060895	3
BP	GO:0007626	locomotory behavior	3/27	0.002849	0.0137018	0.006113	3
BP	GO:0031099	regeneration	3/27	0.002849	0.0137018	0.006113	3
BP	GO:0046890	regulation of lipid biosynthetic	3/27	0.002849	0.0137018	0.006113	3
BP	GO:0043030	regulation of macrophage activation	2/27	0.002853	0.0137018	0.006113	2
BP	GO:0046456	icosanoid biosynthetic process	2/27	0.002853	0.0137018	0.006113	2
BP	GO:0034341	response to interferon-gamma	3/27	0.002889	0.0138152	0.0061636	3
BP	GO:0051222	positive regulation of protein transport	4/27	0.00289	0.0138152	0.0061636	4
BP	GO:0022029	telencephalon cell migration	2/27	0.002956	0.014063	0.0062741	2
BP	GO:1903078	positive regulation of protein localization to plasma membrane	2/27	0.002956	0.014063	0.0062741	2
BP	GO:0002066	columnar/cuboidal epithelial cell development	2/27	0.003061	0.0144932	0.0064661	2
BP	GO:1903428	positive regulation of reactive oxygen species biosynthetic process	2/27	0.003061	0.0144932	0.0064661	2
BP	GO:0034764	positive regulation of transmembrane transport	2/27	0.003099	0.0146064	0.0065166	3
BP	GO:0035637	multicellular organismal signaling	3/27	0.003099	0.0146064	0.0065166	3
BP	GO:0048568	embryonic organ development	4/27	0.003147	0.0147897	0.0065983	4
BP	GO:0019229	regulation of vasoconstriction	2/27	0.003167	0.0147897	0.0065983	2
BP	GO:0030520	intracellular estrogen receptor signaling pathway	2/27	0.003167	0.0147897	0.0065983	2
BP	GO:0061900	glial cell activation	2/27	0.003167	0.0147897	0.0065983	2
BP	GO:0090276	regulation of peptide hormone	3/27	0.003273	0.0151204	0.0067459	3
BP	GO:0010518	positive regulation of phospholipase activity	2/27	0.003275	0.0151204	0.0067459	2
BP	GO:0021885	forebrain cell migration	2/27	0.003275	0.0151204	0.0067459	2
BP	GO:0032890	regulation of organic acid transport	2/27	0.003275	0.0151204	0.0067459	2
BP	GO:0045824	negative regulation of innate immune response	2/27	0.003275	0.0151204	0.0067459	2
BP	GO:0006816	calcium ion transport	4/27	0.003308	0.0152357	0.0067973	4
BP	GO:0006006	glucose metabolic process	3/27	0.003318	0.0152464	0.0068021	3
BP	GO:0009612	response to mechanical stimulus	3/27	0.003363	0.0154174	0.0068784	3

BP	GO:1902808	positive regulation of cell cycle G1/S phase transition	2/27	0.003385	0.0154864	0.0069092	2
BP	GO:1903531	negative regulation of secretion by cell	3/27	0.003408	0.0155543	0.0069395	3
BP	GO:0019932	second-messenger-mediated signaling	4/27	0.003446	0.0156953	0.0070024	4
BP	GO:0032370	positive regulation of lipid transport	2/27	0.003497	0.0158893	0.0070889	2
BP	GO:0019935	cyclic-nucleotide-mediated signaling	3/27	0.003592	0.0162207	0.0072368	3
BP	GO:0070374	positive regulation of ERK1 and ERK2 cascade	3/27	0.003592	0.0162207	0.0072368	3
BP	GO:0002260	lymphocyte homeostasis	2/27	0.00361	0.0162207	0.0072368	2
BP	GO:0010676	positive regulation of cellular carbohydrate metabolic process	2/27	0.00361	0.0162207	0.0072368	2
BP	GO:0032623	interleukin-2 production	2/27	0.00361	0.0162207	0.0072368	2
BP	GO:0044282	small molecule catabolic process	4/27	0.003618	0.0162207	0.0072368	4
BP	GO:0032869	cellular response to insulin stimulus	3/27	0.00364	0.0162813	0.0072638	3
BP	GO:0032371	regulation of sterol transport	2/27	0.003725	0.0164827	0.0073537	2
BP	GO:0032374	regulation of cholesterol transport	2/27	0.003725	0.0164827	0.0073537	2
BP	GO:0046622	positive regulation of organ growth	2/27	0.003725	0.0164827	0.0073537	2
BP	GO:0046686	response to cadmium ion	2/27	0.003725	0.0164827	0.0073537	2
BP	GO:1904377	positive regulation of protein localization to cell periphery	2/27	0.003725	0.0164827	0.0073537	2
BP	GO:1904951	positive regulation of establishment of protein localization	4/27	0.003947	0.0174273	0.0077751	4
BP	GO:0045444	fat cell differentiation	3/27	0.00398	0.0175324	0.007822	3
BP	GO:0030595	leukocyte chemotaxis	3/27	0.00403	0.0177147	0.0079033	3
BP	GO:0045428	regulation of nitric oxide biosynthetic process	3/27	0.004081	0.0178595	0.0079679	2
BP	GO:1905207	regulation of cardiocyte differentiation	2/27	0.004081	0.0178595	0.0079679	2
BP	GO:0023061	signal release	4/27	0.004135	0.0180604	0.0080575	4
BP	GO:0048662	negative regulation of smooth muscle cell proliferation	2/27	0.004202	0.0183134	0.0081704	2
BP	GO:0003012	muscle system process	4/27	0.004232	0.0184014	0.0082097	4
BP	GO:0046323	glucose import	2/27	0.004326	0.0187706	0.0083744	2
BP	GO:0042698	ovulation cycle	2/27	0.004451	0.0192312	0.0085799	2
BP	GO:0070227	lymphocyte apoptotic process	2/27	0.004451	0.0192312	0.0085799	2
BP	GO:0048738	cardiac muscle tissue development	3/27	0.004499	0.0193963	0.0086536	3
BP	GO:0071453	cellular response to oxygen levels	3/27	0.004553	0.0195289	0.0087127	3
BP	GO:0010517	regulation of phospholipase activity	2/27	0.004578	0.0195289	0.0087127	2
BP	GO:0032720	negative regulation of tumor necrosis factor production	2/27	0.004578	0.0195289	0.0087127	2
BP	GO:0034121	regulation of toll-like receptor signaling pathway	2/27	0.004578	0.0195289	0.0087127	2
BP	GO:0051966	regulation of synaptic transmission, glutamatergic	2/27	0.004578	0.0195289	0.0087127	2
BP	GO:0043122	regulation of I-kappaB kinase/NF-kappaB signaling	3/27	0.004718	0.0200819	0.0089594	3
BP	GO:0051048	negative regulation of secretion	3/27	0.004773	0.0202271	0.0090242	3
BP	GO:0019226	transmission of nerve impulse	2/27	0.004837	0.0202271	0.0090242	2
BP	GO:0032024	positive regulation of insulin secretion	2/27	0.004837	0.0202271	0.0090242	2
BP	GO:0051881	regulation of mitochondrial membrane potential	2/27	0.004837	0.0202271	0.0090242	2
BP	GO:0061035	regulation of cartilage development	2/27	0.004837	0.0202271	0.0090242	2
BP	GO:0061180	mammary gland epithelium	2/27	0.004837	0.0202271	0.0090242	2
BP	GO:1903556	negative regulation of tumor necrosis factor superfamily cytokine	2/27	0.004837	0.0202271	0.0090242	2
BP	GO:0034765	regulation of ion transmembrane transport	4/27	0.004842	0.0202271	0.0090242	4
BP	GO:0070838	divalent metal ion transport	4/27	0.004842	0.0202271	0.0090242	4
BP	GO:0048871	multicellular organismal homeostasis	4/27	0.004913	0.0204825	0.0091382	4
BP	GO:0042593	glucose homeostasis	3/27	0.004943	0.0205633	0.0091742	3
BP	GO:0031100	animal organ regeneration	2/27	0.004969	0.0205867	0.0091847	2
BP	GO:0060350	endochondral bone morphogenesis	2/27	0.004969	0.0205867	0.0091847	2
BP	GO:0033500	carbohydrate homeostasis	3/27	0.005	0.0206739	0.0092235	3
BP	GO:0009266	response to temperature stimulus	3/27	0.005057	0.0208277	0.0092922	3
BP	GO:0072511	divalent inorganic cation transport	4/27	0.005058	0.0208277	0.0092922	4
BP	GO:0051896	regulation of protein kinase B	3/27	0.005115	0.0210233	0.0093794	3

BP	GO:0150076	neuroinflammatory response	2/27	0.005237	0.0214796	0.009583	2
BP	GO:0019318	hexose metabolic process	3/27	0.005412	0.022151	0.0098825	3
BP	GO:0030072	peptide hormone secretion	3/27	0.005472	0.0223533	0.0099728	3
BP	GO:0006809	nitric oxide biosynthetic process	2/27	0.005512	0.0223812	0.0099852	2
BP	GO:0008344	adult locomotory behavior	2/27	0.005512	0.0223812	0.0099852	2
BP	GO:0051937	catecholamine transport	2/27	0.005512	0.0223812	0.0099852	2
BP	GO:0008016	regulation of heart contraction	3/27	0.005533	0.0224208	0.0100029	3
BP	GO:0008306	associative learning	2/27	0.005652	0.0227823	0.0101642	2
BP	GO:0055021	regulation of cardiac muscle tissue growth	2/27	0.005652	0.0227823	0.0101642	2
BP	GO:2000027	regulation of animal organ morphogenesis	3/27	0.005656	0.0227823	0.0101642	3
BP	GO:0051924	regulation of calcium ion transport	3/27	0.005718	0.0229867	0.0102554	3
BP	GO:0071260	cellular response to mechanical	2/27	0.005794	0.0232449	0.0103706	2
BP	GO:0014855	striated muscle cell proliferation	2/27	0.006082	0.0243046	0.0108434	2
BP	GO:0048708	astrocyte differentiation	2/27	0.006082	0.0243046	0.0108434	2
BP	GO:0032642	regulation of chemokine production	2/27	0.006229	0.0246949	0.0110175	2
BP	GO:0046209	nitric oxide metabolic process	2/27	0.006229	0.0246949	0.0110175	2
BP	GO:0051279	regulation of release of sequestered calcium ion into cytosol	2/27	0.006229	0.0246949	0.0110175	2
BP	GO:1905954	positive regulation of lipid	2/27	0.006229	0.0246949	0.0110175	2
BP	GO:0045913	positive regulation of carbohydrate metabolic process	2/27	0.006377	0.0252327	0.0112575	2
BP	GO:0046883	regulation of hormone secretion	3/27	0.006496	0.025654	0.0114454	3
BP	GO:0060420	regulation of heart growth	2/27	0.006527	0.0257246	0.0114769	2
BP	GO:0002028	regulation of sodium ion transport	2/27	0.006678	0.0261021	0.0116453	2
BP	GO:1904705	regulation of vascular smooth muscle cell proliferation	2/27	0.006678	0.0261021	0.0116453	2
BP	GO:1990874	vascular smooth muscle cell proliferation	2/27	0.006678	0.0261021	0.0116453	2
BP	GO:2001057	reactive nitrogen species metabolic process	2/27	0.006678	0.0261021	0.0116453	2
BP	GO:0007249	I-kappaB kinase/NF-kappaB signaling	3/27	0.0067	0.0261021	0.0116453	3
BP	GO:0043491	protein kinase B signaling	3/27	0.0067	0.0261021	0.0116453	3
BP	GO:0001776	leukocyte homeostasis	2/27	0.006831	0.0265104	0.0118275	2
BP	GO:0042058	regulation of epidermal growth factor receptor signaling pathway	2/27	0.006831	0.0265104	0.0118275	2
BP	GO:0001892	embryonic placenta development	2/27	0.006985	0.0270582	0.0120719	2
BP	GO:0016054	organic acid catabolic process	3/27	0.007119	0.0274702	0.0122557	3
BP	GO:0046395	carboxylic acid catabolic process	3/27	0.007119	0.0274702	0.0122557	3
BP	GO:0032602	chemokine production	2/27	0.007299	0.0280054	0.0124945	2
BP	GO:0097306	cellular response to alcohol	2/27	0.007299	0.0280054	0.0124945	2
BP	GO:1901019	regulation of calcium ion transmembrane transporter activity	2/27	0.007299	0.0280054	0.0124945	2
BP	GO:0001843	neural tube closure	2/27	0.007458	0.0285083	0.0127188	2
BP	GO:0015844	monoamine transport	2/27	0.007458	0.0285083	0.0127188	2
BP	GO:0060047	heart contraction	3/27	0.00748	0.0285363	0.0127313	3
BP	GO:0060606	tube closure	2/27	0.007619	0.0289589	0.0129199	2
BP	GO:1901992	positive regulation of mitotic cell cycle phase transition	2/27	0.007619	0.0289589	0.0129199	2
BP	GO:0032409	regulation of transporter activity	3/27	0.007702	0.0292169	0.013035	3
BP	GO:0045833	negative regulation of lipid metabolic process	2/27	0.007782	0.0294654	0.0131458	2
BP	GO:0035249	synaptic transmission, glutamatergic	2/27	0.007946	0.029974	0.0133728	2
BP	GO:1901184	regulation of ERBB signaling pathway	2/27	0.007946	0.029974	0.0133728	2
BP	GO:0003015	heart process	3/27	0.008234	0.0309974	0.0138294	3
BP	GO:0044070	regulation of anion transport	2/27	0.008278	0.0309974	0.0138294	2
BP	GO:0051952	regulation of amine transport	2/27	0.008278	0.0309974	0.0138294	2
BP	GO:1903076	regulation of protein localization to plasma membrane	2/27	0.008278	0.0309974	0.0138294	2
BP	GO:0071356	cellular response to tumor necrosis factor	3/27	0.008312	0.0310658	0.0138598	3
BP	GO:0005996	monosaccharide metabolic process	3/27	0.00839	0.0313004	0.0139645	3
BP	GO:0042632	cholesterol homeostasis	2/27	0.008447	0.0314541	0.0140331	2
BP	GO:0051146	striated muscle cell differentiation	3/27	0.008469	0.0314777	0.0140436	3

BP	GO:0014020	primary neural tube formation	2/27	0.008617	0.0318532	0.0142112	2
BP	GO:0043255	regulation of carbohydrate biosynthetic process	2/27	0.008617	0.0318532	0.0142112	2
BP	GO:0055092	sterol homeostasis	2/27	0.008617	0.0318532	0.0142112	2
BP	GO:0050764	regulation of phagocytosis	2/27	0.008789	0.0323696	0.0144416	2
BP	GO:2001243	negative regulation of intrinsic apoptotic signaling pathway	2/27	0.008789	0.0323696	0.0144416	2
BP	GO:1901570	fatty acid derivative biosynthetic process	2/27	0.008962	0.0329478	0.0146995	2
BP	GO:0070372	regulation of ERK1 and ERK2	3/27	0.009032	0.033144	0.014787	3
BP	GO:0022600	digestive system process	2/27	0.009137	0.0332279	0.0148244	2
BP	GO:0030301	cholesterol transport	2/27	0.009137	0.0332279	0.0148244	2
BP	GO:0055024	regulation of cardiac muscle tissue development	2/27	0.009137	0.0332279	0.0148244	2
BP	GO:0060079	excitatory postsynaptic potential	2/27	0.009137	0.0332279	0.0148244	2
BP	GO:2001023	regulation of response to drug	2/27	0.009137	0.0332279	0.0148244	2
BP	GO:0002027	regulation of heart rate	2/27	0.009313	0.0337477	0.0150564	2
BP	GO:1903426	regulation of reactive oxygen species biosynthetic process	2/27	0.009313	0.0337477	0.0150564	2
BP	GO:0060326	cell chemotaxis	3/27	0.009363	0.033808	0.0150833	3
BP	GO:1903037	regulation of leukocyte cell-cell adhesion	3/27	0.009363	0.033808	0.0150833	3
BP	GO:0000079	regulation of cyclin-dependent protein serine/threonine kinase activity	2/27	0.009491	0.0340867	0.0152076	2
BP	GO:0010522	regulation of calcium ion transport into cytosol	2/27	0.009491	0.0340867	0.0152076	2
BP	GO:0015837	amine transport	2/27	0.009491	0.0340867	0.0152076	2
BP	GO:0018958	phenol-containing compound metabolic process	2/27	0.00967	0.0345468	0.0154129	2
BP	GO:0032091	negative regulation of protein binding	2/27	0.00967	0.0345468	0.0154129	2
BP	GO:0034766	negative regulation of ion transmembrane transport	2/27	0.00967	0.0345468	0.0154129	2
BP	GO:0009895	negative regulation of catabolic	3/27	0.009702	0.0345978	0.0154356	3
BP	GO:0071887	leukocyte apoptotic process	2/27	0.009851	0.0350691	0.0156459	2
BP	GO:0001841	neural tube formation	2/27	0.010034	0.0353934	0.0157906	2
BP	GO:0006641	triglyceride metabolic process	2/27	0.010034	0.0353934	0.0157906	2
BP	GO:0008593	regulation of Notch signaling pathway	2/27	0.010034	0.0353934	0.0157906	2
BP	GO:0055017	cardiac muscle tissue growth	2/27	0.010034	0.0353934	0.0157906	2
BP	GO:0034612	response to tumor necrosis factor	3/27	0.010047	0.0353934	0.0157906	3
BP	GO:0046879	hormone secretion	3/27	0.010047	0.0353934	0.0157906	3
BP	GO:1901214	regulation of neuron death	3/27	0.010135	0.0356397	0.0159005	3
BP	GO:0042136	neurotransmitter biosynthetic process	2/27	0.010218	0.0357007	0.0159277	2
BP	GO:1901989	positive regulation of cell cycle phase transition	2/27	0.010218	0.0357007	0.0159277	2
BP	GO:1904029	regulation of cyclin-dependent protein kinase activity	2/27	0.010218	0.0357007	0.0159277	2
BP	GO:0009416	response to light stimulus	3/27	0.010223	0.0357007	0.0159277	3
BP	GO:0070371	ERK1 and ERK2 cascade	3/27	0.01049	0.0365686	0.0163149	3
BP	GO:0015696	ammonium transport	2/27	0.01059	0.0368547	0.0164426	2
BP	GO:0001676	long-chain fatty acid metabolic	2/27	0.010778	0.0374459	0.0167063	2
BP	GO:0007568	aging	3/27	0.010851	0.0375708	0.016762	3
BP	GO:0071375	cellular response to peptide hormone stimulus	3/27	0.010851	0.0375708	0.016762	3
BP	GO:0009914	hormone transport	3/27	0.010943	0.0377585	0.0168458	3
BP	GO:0060562	epithelial tube morphogenesis	3/27	0.010943	0.0377585	0.0168458	3
BP	GO:0016101	diterpenoid metabolic process	2/27	0.010968	0.037781	0.0168558	2
BP	GO:0002065	columnar/cuboidal epithelial cell differentiation	2/27	0.011352	0.0388397	0.0173281	2
BP	GO:0015918	sterol transport	2/27	0.011352	0.0388397	0.0173281	2
BP	GO:0060419	heart growth	2/27	0.011352	0.0388397	0.0173281	2
BP	GO:1903510	mucopolysaccharide metabolic	2/27	0.011352	0.0388397	0.0173281	2
BP	GO:0001655	urogenital system development	3/27	0.011692	0.0399337	0.0178162	3
BP	GO:0060349	bone morphogenesis	2/27	0.011742	0.0399902	0.0178414	2
BP	GO:0008202	steroid metabolic process	3/27	0.011787	0.0399902	0.0178414	3
BP	GO:0071214	cellular response to abiotic stimulus	3/27	0.011787	0.0399902	0.0178414	3

BP	GO:0104004	cellular response to environmental stimulus	3/27	0.011787	0.0399902	0.0178414	3
BP	GO:0032963	collagen metabolic process	2/27	0.01194	0.040371	0.0180113	2
BP	GO:1904375	regulation of protein localization to cell periphery	2/27	0.01194	0.040371	0.0180113	2
BP	GO:0051251	positive regulation of lymphocyte activation	3/27	0.012077	0.0407678	0.0181884	3
BP	GO:0007613	memory	2/27	0.012338	0.0415115	0.0185202	2
BP	GO:0010906	regulation of glucose metabolic	2/27	0.012338	0.0415115	0.0185202	2
BP	GO:0007159	leukocyte cell-cell adhesion	3/27	0.012371	0.0415515	0.018538	3
BP	GO:0007173	epidermal growth factor receptor signaling pathway	2/27	0.012743	0.0427308	0.0190642	2
BP	GO:1904062	regulation of cation transmembrane transport	3/27	0.01287	0.0430839	0.0192217	3
BP	GO:0006721	terpenoid metabolic process	2/27	0.012948	0.0432731	0.0193061	2
BP	GO:0010721	negative regulation of cell	3/27	0.013073	0.0436182	0.01946	3
BP	GO:1903409	reactive oxygen species biosynthetic process	2/27	0.013361	0.0444343	0.0198241	2
BP	GO:1905477	positive regulation of protein localization to membrane	2/27	0.013361	0.0444343	0.0198241	2
BP	GO:0001838	embryonic epithelial tube formation	2/27	0.01378	0.0456577	0.02037	2
BP	GO:0014066	regulation of phosphatidylinositol 3-kinase signaling	2/27	0.01378	0.0456577	0.02037	2
BP	GO:0010631	epithelial cell migration	3/27	0.013797	0.0456577	0.02037	3
BP	GO:0045471	response to ethanol	2/27	0.013992	0.0459067	0.0204811	2
BP	GO:0090132	epithelium migration	3/27	0.014114	0.0459067	0.0204811	3
BP	GO:0001660	fever generation	1/27	0.014371	0.0459067	0.0204811	1
BP	GO:0010739	positive regulation of protein kinase A signaling	1/27	0.014371	0.0459067	0.0204811	1
BP	GO:0010749	regulation of nitric oxide mediated signal transduction	1/27	0.014371	0.0459067	0.0204811	1
BP	GO:0010918	positive regulation of mitochondrial membrane potential	1/27	0.014371	0.0459067	0.0204811	1
BP	GO:0030432	peristalsis	1/27	0.014371	0.0459067	0.0204811	1
BP	GO:0032000	positive regulation of fatty acid beta-oxidation	1/27	0.014371	0.0459067	0.0204811	1
BP	GO:0032070	regulation of deoxyribonuclease	1/27	0.014371	0.0459067	0.0204811	1
BP	GO:0032308	positive regulation of prostaglandin secretion	1/27	0.014371	0.0459067	0.0204811	1
BP	GO:0045713	low-density lipoprotein particle receptor biosynthetic process	1/27	0.014371	0.0459067	0.0204811	1
BP	GO:0045792	negative regulation of cell size	1/27	0.014371	0.0459067	0.0204811	1
BP	GO:0046322	negative regulation of fatty acid oxidation	1/27	0.014371	0.0459067	0.0204811	1
BP	GO:0046532	regulation of photoreceptor cell differentiation	1/27	0.014371	0.0459067	0.0204811	1
BP	GO:0048149	behavioral response to ethanol	1/27	0.014371	0.0459067	0.0204811	1
BP	GO:0051006	positive regulation of lipoprotein lipase activity	1/27	0.014371	0.0459067	0.0204811	1
BP	GO:0060068	vagina development	1/27	0.014371	0.0459067	0.0204811	1
BP	GO:0060456	positive regulation of digestive system process	1/27	0.014371	0.0459067	0.0204811	1
BP	GO:0140052	cellular response to oxidised low-density lipoprotein particle stimulus	1/27	0.014371	0.0459067	0.0204811	1
BP	GO:1902033	regulation of hematopoietic stem cell proliferation	1/27	0.014371	0.0459067	0.0204811	1
BP	GO:1903799	negative regulation of production of miRNAs involved in gene silencing by miRNA	1/27	0.014371	0.0459067	0.0204811	1
BP	GO:1903800	positive regulation of production of miRNAs involved in gene silencing by miRNA	1/27	0.014371	0.0459067	0.0204811	1
BP	GO:0035270	endocrine system development	2/27	0.014419	0.0459874	0.0205171	2
BP	GO:0034763	negative regulation of transmembrane transport	2/27	0.014635	0.0465824	0.0207825	2

BP	GO:0001666	response to hypoxia	3/27	0.014652	0.0465824	0.0207825	3
BP	GO:0006936	muscle contraction	3/27	0.014761	0.0467819	0.0208715	3
BP	GO:0090130	tissue migration	3/27	0.014761	0.0467819	0.0208715	3
BP	GO:0006639	acylglycerol metabolic process	2/27	0.014853	0.0469251	0.0209354	2
BP	GO:1903038	negative regulation of leukocyte cell-cell adhesion	2/27	0.014853	0.0469251	0.0209354	2
BP	GO:0006638	neutral lipid metabolic process	2/27	0.015071	0.0475337	0.0212069	2
BP	GO:0006066	alcohol metabolic process	3/27	0.015202	0.0475337	0.0212069	3
BP	GO:0030518	intracellular steroid hormone receptor signaling pathway	2/27	0.015513	0.0475337	0.0212069	2
BP	GO:0055007	cardiac muscle cell differentiation	2/27	0.015513	0.0475337	0.0212069	2
BP	GO:0072175	epithelial tube formation	2/27	0.015513	0.0475337	0.0212069	2
BP	GO:0001508	action potential	2/27	0.015736	0.0475337	0.0212069	2
BP	GO:0035690	cellular response to drug	3/27	0.015763	0.0475337	0.0212069	3
BP	GO:0003264	regulation of cardioblast proliferation	1/27	0.015798	0.0475337	0.0212069	1
BP	GO:0007195	adenylate cyclase-inhibiting dopamine receptor signaling pathway	1/27	0.015798	0.0475337	0.0212069	1
BP	GO:0019371	cyclooxygenase pathway	1/27	0.015798	0.0475337	0.0212069	1
BP	GO:0021892	cerebral cortex GABAergic interneuron differentiation	1/27	0.015798	0.0475337	0.0212069	1
BP	GO:0032306	regulation of prostaglandin secretion	1/27	0.015798	0.0475337	0.0212069	1
BP	GO:0033860	regulation of NAD(P)H oxidase	1/27	0.015798	0.0475337	0.0212069	1
BP	GO:0034776	response to histamine	1/27	0.015798	0.0475337	0.0212069	1
BP	GO:0035404	histone-serine phosphorylation	1/27	0.015798	0.0475337	0.0212069	1
BP	GO:0042670	retinal cone cell differentiation	1/27	0.015798	0.0475337	0.0212069	1
BP	GO:0045628	regulation of T-helper 2 cell differentiation	1/27	0.015798	0.0475337	0.0212069	1
BP	GO:0045899	positive regulation of RNA polymerase II transcriptional preinitiation complex assembly	1/27	0.015798	0.0475337	0.0212069	1
BP	GO:0046549	retinal cone cell development	1/27	0.015798	0.0475337	0.0212069	1
BP	GO:0048548	regulation of pinocytosis	1/27	0.015798	0.0475337	0.0212069	1
BP	GO:0051974	negative regulation of telomerase activity	1/27	0.015798	0.0475337	0.0212069	1
BP	GO:0060159	regulation of dopamine receptor signaling pathway	1/27	0.015798	0.0475337	0.0212069	1
BP	GO:0060525	prostate glandular acinus development	1/27	0.015798	0.0475337	0.0212069	1
BP	GO:0060736	prostate gland growth	1/27	0.015798	0.0475337	0.0212069	1
BP	GO:0060856	establishment of blood-brain barrier	1/27	0.015798	0.0475337	0.0212069	1
BP	GO:0061365	positive regulation of triglyceride lipase activity	1/27	0.015798	0.0475337	0.0212069	1
BP	GO:0070673	response to interleukin-18	1/27	0.015798	0.0475337	0.0212069	1
BP	GO:0070857	regulation of bile acid biosynthetic process	1/27	0.015798	0.0475337	0.0212069	1
BP	GO:0086023	adenylate cyclase-activating adrenergic receptor signaling pathway involved in heart process	1/27	0.015798	0.0475337	0.0212069	1
BP	GO:0099149	regulation of postsynaptic neurotransmitter receptor	1/27	0.015798	0.0475337	0.0212069	1
BP	GO:1901077	regulation of relaxation of muscle	1/27	0.015798	0.0475337	0.0212069	1
BP	GO:1904468	negative regulation of tumor necrosis factor secretion	1/27	0.015798	0.0475337	0.0212069	1
BP	GO:0032535	regulation of cellular component size	3/27	0.015877	0.0476304	0.02125	3
BP	GO:0036293	response to decreased oxygen levels	3/27	0.015877	0.0476304	0.02125	3
BP	GO:0006766	vitamin metabolic process	2/27	0.01596	0.0478098	0.0213301	2
BP	GO:0001889	liver development	2/27	0.016186	0.0484142	0.0215997	2
BP	GO:0008584	male gonad development	2/27	0.016871	0.0496752	0.0221623	2
BP	GO:0061008	hepaticobiliary system development	2/27	0.016871	0.0496752	0.0221623	2
BP	GO:0002696	positive regulation of leukocyte activation	3/27	0.017039	0.0496752	0.0221623	3
BP	GO:0006720	isoprenoid metabolic process	2/27	0.017102	0.0496752	0.0221623	2
BP	GO:0007586	digestion	2/27	0.017102	0.0496752	0.0221623	2
BP	GO:0046546	development of primary male sexual characteristics	2/27	0.017102	0.0496752	0.0221623	2
BP	GO:0051588	regulation of neurotransmitter	2/27	0.017102	0.0496752	0.0221623	2

BP	GO:0002024	diet induced thermogenesis	1/27	0.017222	0.0496752	0.0221623	1
BP	GO:0002674	negative regulation of acute inflammatory response	1/27	0.017222	0.0496752	0.0221623	1
BP	GO:0002903	negative regulation of B cell apoptotic process	1/27	0.017222	0.0496752	0.0221623	1
BP	GO:0006068	ethanol catabolic process	1/27	0.017222	0.0496752	0.0221623	1
BP	GO:0006069	ethanol oxidation	1/27	0.017222	0.0496752	0.0221623	1
BP	GO:0010649	regulation of cell communication by electrical coupling	1/27	0.017222	0.0496752	0.0221623	1
BP	GO:0010960	magnesium ion homeostasis	1/27	0.017222	0.0496752	0.0221623	1
BP	GO:0030157	pancreatic juice secretion	1/27	0.017222	0.0496752	0.0221623	1
BP	GO:0032429	regulation of phospholipase A2	1/27	0.017222	0.0496752	0.0221623	1
BP	GO:0033197	response to vitamin E	1/27	0.017222	0.0496752	0.0221623	1
BP	GO:0060442	branching involved in prostate gland morphogenesis	1/27	0.017222	0.0496752	0.0221623	1
BP	GO:0060453	regulation of gastric acid secretion	1/27	0.017222	0.0496752	0.0221623	1
BP	GO:0071073	positive regulation of phospholipid biosynthetic process	1/27	0.017222	0.0496752	0.0221623	1
BP	GO:0071468	cellular response to acidic pH	1/27	0.017222	0.0496752	0.0221623	1
BP	GO:1900402	regulation of carbohydrate metabolic process by regulation of transcription from RNA polymerase II promoter	1/27	0.017222	0.0496752	0.0221623	1
BP	GO:1905461	positive regulation of vascular associated smooth muscle cell apoptotic process	1/27	0.017222	0.0496752	0.0221623	1
BP	GO:1905522	negative regulation of macrophage migration	1/27	0.017222	0.0496752	0.0221623	1
BP	GO:2000341	regulation of chemokine (C-X-C motif) ligand 2 production	1/27	0.017222	0.0496752	0.0221623	1
CC	GO:0090575	RNA polymerase II transcription factor complex	8/28	4.96E-11	4.16E-09	3.39E-09	8
CC	GO:0044798	nuclear transcription factor complex	8/28	2.65E-10	1.11E-08	9.06E-09	8
CC	GO:0005667	transcription factor complex	8/28	2.87E-08	8.05E-07	6.55E-07	8
CC	GO:0000790	nuclear chromatin	7/28	7.40E-07	1.55E-05	1.27E-05	7
MF	GO:0004879	nuclear receptor activity	10/28	7.93E-20	7.21E-18	4.09E-18	10
MF	GO:0098531	transcription factor activity, direct ligand regulated sequence-specific DNA binding	10/28	7.93E-20	7.21E-18	4.09E-18	10
MF	GO:0003707	steroid hormone receptor activity	10/28	5.41E-19	3.28E-17	1.86E-17	10
MF	GO:0030374	nuclear receptor transcription coactivator activity	8/28	8.02E-14	3.65E-12	2.07E-12	8
MF	GO:0003713	transcription coactivator activity	9/28	9.18E-10	3.34E-08	1.89E-08	9
MF	GO:0033293	monocarboxylic acid binding	5/28	4.86E-08	1.47E-06	8.36E-07	5
MF	GO:0005496	steroid binding	5/28	3.57E-07	9.29E-06	5.26E-06	5
MF	GO:0004952	dopamine neurotransmitter receptor activity	3/28	4.23E-07	9.61E-06	5.45E-06	3
MF	GO:0035240	dopamine binding	3/28	1.00E-06	2.03E-05	1.15E-05	3
MF	GO:0046965	retinoid X receptor binding	3/28	2.38E-06	4.33E-05	2.45E-05	3
MF	GO:1901338	catecholamine binding	3/28	3.97E-06	6.57E-05	3.72E-05	3
MF	GO:0042974	retinoic acid receptor binding	3/28	9.00E-06	0.0001365	7.74E-05	3
MF	GO:0031406	carboxylic acid binding	5/28	1.17E-05	0.0001644	9.32E-05	5
MF	GO:0043177	organic acid binding	5/28	1.57E-05	0.0002043	0.0001158	5
MF	GO:0042562	hormone binding	4/28	1.91E-05	0.0002323	0.0001317	4
MF	GO:0005504	fatty acid binding	3/28	2.05E-05	0.0002336	0.0001324	3
MF	GO:0001103	RNA polymerase II repressing transcription factor binding	3/28	2.24E-05	0.0002403	0.0001362	3
MF	GO:0035257	nuclear hormone receptor binding	4/28	9.12E-05	0.0009223	0.0005228	4
MF	GO:0001085	RNA polymerase II transcription factor binding	4/28	9.84E-05	0.0009425	0.0005342	4
MF	GO:0070491	repressing transcription factor binding	3/28	0.000189	0.0016091	0.000912	3
MF	GO:0019902	phosphatase binding	4/28	0.000195	0.0016091	0.000912	4
MF	GO:0051427	hormone receptor binding	4/28	0.000195	0.0016091	0.000912	4
MF	GO:0036041	long-chain fatty acid binding	2/28	0.000217	0.001683	0.0009539	2
MF	GO:0070405	ammonium ion binding	3/28	0.000222	0.001683	0.0009539	3
MF	GO:0001223	transcription coactivator binding	2/28	0.000598	0.0043558	0.0024689	2

MF	GO:0001965	G-protein alpha-subunit binding	2/28	0.000652	0.0045646	0.0025872	2
MF	GO:0030594	neurotransmitter receptor activity	3/28	0.000818	0.0055126	0.0031245	3
MF	GO:0004713	protein tyrosine kinase activity	3/28	0.001211	0.0078695	0.0044604	3
MF	GO:0019903	protein phosphatase binding	3/28	0.001373	0.0086188	0.0048851	3
MF	GO:0051393	alpha-actinin binding	2/28	0.001554	0.0094253	0.0053423	2
MF	GO:0030331	estrogen receptor binding	2/28	0.001999	0.0117344	0.0066511	2
MF	GO:0001221	transcription cofactor binding	2/28	0.002094	0.0119106	0.0067509	2
MF	GO:0042805	actinin binding	2/28	0.002393	0.0131993	0.0074814	2
MF	GO:0005516	calmodulin binding	3/28	0.003783	0.0202485	0.0114769	3
MF	GO:0016655	oxidoreductase activity, acting on NAD(P)H, quinone or similar compound as acceptor	2/28	0.004037	0.020994	0.0118994	2
MF	GO:0004714	transmembrane receptor protein tyrosine kinase activity	2/28	0.004305	0.0217636	0.0123357	2
MF	GO:0003714	transcription corepressor activity	3/28	0.006139	0.0301974	0.0171159	3
MF	GO:0001227	DNA-binding transcription repressor activity, RNA polymerase II-specific	3/28	0.006428	0.0307868	0.01745	3
MF	GO:0019199	transmembrane receptor protein kinase activity	2/28	0.006899	0.0321626	0.0182298	2
MF	GO:0051117	ATPase binding	2/28	0.007069	0.0321626	0.0182298	2
MF	GO:0051219	phosphoprotein binding	2/28	0.00759	0.0336925	0.019097	2
MF	GO:0035258	steroid hormone receptor binding	2/28	0.009255	0.0401052	0.0227317	2

Table S3 KEGG Pathway Enrichment Analyses of potential targets

ID	Description	GeneRatio	pvalue	p.adjust	qvalue	Count
hsa05221	Acute myeloid leukemia	4/25	4.89E-05	0.003994	0.002999	4
hsa04915	Estrogen signaling pathway	5/25	5.59E-05	0.003994	0.002999	5
hsa03320	PPAR signaling pathway	4/25	8.45E-05	0.004027	0.003024	4
hsa01522	Endocrine resistance	4/25	0.000216	0.007716	0.005793	4
hsa04024	cAMP signaling pathway	5/25	0.000458	0.01167	0.008762	5
hsa04080	Ieuroactive ligand-receptor interactio	6/25	0.00049	0.01167	0.008762	6
hsa04728	Dopaminergic synapse	4/25	0.000672	0.013733	0.010311	4
hsa05224	Breast cancer	4/25	0.001007	0.01648	0.012374	4
hsa05223	Non-small cell lung cancer	3/25	0.001068	0.01648	0.012374	3
hsa05160	Hepatitis C	4/25	0.001226	0.01648	0.012374	4
hsa04917	Prolactin signaling pathway	3/25	0.001268	0.01648	0.012374	3
hsa05235	ression and PD-1 checkpoint pathwa	3/25	0.00253	0.030144	0.022633	3
hsa04931	Insulin resistance	3/25	0.004374	0.048113	0.036125	3
hsa04919	Thyroid hormone signaling pathway	3/25	0.005736	0.058588	0.04399	3
hsa04932	n-alcoholic fatty liver disease (NAFI	3/25	0.010645	0.101479	0.076193	3
hsa04923	Regulation of lipolysis in adipocytes	2/25	0.012904	0.109619	0.082305	2
hsa04630	JAK-STAT signaling pathway	3/25	0.013345	0.109619	0.082305	3
hsa05213	Endometrial cancer	2/25	0.013798	0.109619	0.082305	2
hsa00561	Glycerolipid metabolism	2/25	0.015189	0.114318	0.085833	2
hsa04929	GnRH secretion	2/25	0.016638	0.118963	0.089321	2
hsa04920	Adipocytokine signaling pathway	2/25	0.019179	0.121532	0.091249	2
hsa05230	Central carbon metabolism in cancer	2/25	0.019179	0.121532	0.091249	2
hsa04976	Bile secretion	2/25	0.020778	0.121532	0.091249	2
hsa05218	Melanoma	2/25	0.020778	0.121532	0.091249	2
hsa04020	Calcium signaling pathway	3/25	0.021247	0.121532	0.091249	3
hsa05214	Glioma	2/25	0.022431	0.121782	0.091437	2
hsa05212	Pancreatic cancer	2/25	0.022994	0.121782	0.091437	2
hsa01521	3FR tyrosine kinase inhibitor resistar	2/25	0.024717	0.122634	0.092077	2
hsa05205	Proteoglycans in cancer	3/25	0.02487	0.122634	0.092077	3
hsa04012	ErbB signaling pathway	2/25	0.028318	0.133485	0.100224	2
hsa05210	Colorectal cancer	2/25	0.028937	0.133485	0.100224	2
hsa04540	Gap junction	2/25	0.030193	0.133592	0.100305	2
hsa04211	Longevity regulating pathway	2/25	0.030829	0.133592	0.100305	2
hsa05032	Morphine addiction	2/25	0.032117	0.13508	0.101422	2
hsa04666	Fc gamma R-mediated phagocytosis	2/25	0.033426	0.136569	0.10254	2
hsa05215	Prostate cancer	2/25	0.036107	0.142186	0.106758	2
hsa05231	Choline metabolism in cancer	2/25	0.036789	0.142186	0.106758	2
hsa04933	3E signaling pathway in diabetic con	2/25	0.03817	0.143641	0.10785	2
hsa04922	Glucagon signaling pathway	2/25	0.042431	0.151693	0.113895	2
hsa04928	roid hormone synthesis, secretion an	2/25	0.042431	0.151693	0.113895	2
hsa04066	HIF-1 signaling pathway	2/25	0.044627	0.15565	0.116866	2
hsa00360	Phenylalanine metabolism	1/25	0.051622	0.175762	0.131967	1
hsa04152	AMPK signaling pathway	2/25	0.053029	0.176351	0.132409	2
hsa04380	Osteoclast differentiation	2/25	0.059469	0.191595	0.143855	2
hsa04926	Relaxin signaling pathway	2/25	0.060292	0.191595	0.143855	2
hsa04068	FoxO signaling pathway	2/25	0.061951	0.192587	0.1446	2
hsa00340	Histidine metabolism	1/25	0.069224	0.210616	0.158137	1
hsa04072	Phospholipase D signaling pathway	2/25	0.076669	0.222811	0.167293	2
hsa05226	Gastric cancer	2/25	0.077567	0.222811	0.167293	2
hsa00790	Folate biosynthesis	1/25	0.077906	0.222811	0.167293	1
hsa00053	Ascorbate and aldarate metabolism	1/25	0.080783	0.226509	0.170069	1
hsa00410	beta-Alanine metabolism	1/25	0.092204	0.248778	0.18679	1
hsa01523	Antifolate resistance	1/25	0.092204	0.248778	0.18679	1

hsa05225	Hepatocellular carcinoma	2/25	0.095256	0.252252	0.189398	2
hsa00350	Tyrosine metabolism	1/25	0.10629	0.276354	0.207494	1
hsa05216	Thyroid cancer	1/25	0.109082	0.276535	0.20763	1
hsa05202	'ranscriptional misregulation in cance	2/25	0.112991	0.276535	0.20763	2
hsa00620	Pyruvate metabolism	1/25	0.11464	0.276535	0.20763	1
hsa04062	Chemokine signaling pathway	2/25	0.116029	0.276535	0.20763	2
hsa05167	si sarcoma-associated herpesvirus inf	2/25	0.116029	0.276535	0.20763	2
hsa05219	Bladder cancer	1/25	0.120165	0.281699	0.211508	1
hsa00380	Tryptophan metabolism	1/25	0.122916	0.283499	0.212859	1
hsa04510	Focal adhesion	2/25	0.126308	0.286698	0.215261	2
hsa00071	Fatty acid degradation	1/25	0.128391	0.286874	0.215393	1
hsa02010	ABC transporters	1/25	0.131117	0.288457	0.216581	1
hsa04973	Carbohydrate digestion and absorptio	1/25	0.136543	0.292822	0.219859	1
hsa04015	Rap1 signaling pathway	2/25	0.137866	0.292822	0.219859	2
hsa00280	line, leucine and isoleucine degradat	1/25	0.139244	0.292822	0.219859	1
hsa05030	Cocaine addiction	1/25	0.141937	0.294159	0.220863	1
hsa00330	Arginine and proline metabolism	1/25	0.144622	0.295442	0.221826	1
hsa04961	and other factor-regulated calcium re	1/25	0.152628	0.305862	0.22965	1
hsa05163	Human cytomegalovirus infection	2/25	0.154001	0.305862	0.22965	2
hsa04714	Thermogenesis	2/25	0.160562	0.312398	0.234558	2
hsa04014	Ras signaling pathway	2/25	0.161661	0.312398	0.234558	2
hsa04370	VEGF signaling pathway	1/25	0.168425	0.318729	0.239311	1
hsa05131	Shigellosis	2/25	0.169395	0.318729	0.239311	2
hsa00310	Lysine degradation	1/25	0.173627	0.322451	0.242105	1
hsa04213	evity regulating pathway - multiple sp	1/25	0.176217	0.323064	0.242566	1
hsa00590	Arachidonic acid metabolism	1/25	0.178799	0.323648	0.243004	1
hsa00010	Glycolysis / Gluconeogenesis	1/25	0.191591	0.334459	0.251122	1
hsa04664	Fc epsilon RI signaling pathway	1/25	0.191591	0.334459	0.251122	1
hsa05031	Amphetamine addiction	1/25	0.194127	0.334459	0.251122	1
hsa05211	Renal cell carcinoma	1/25	0.194127	0.334459	0.251122	1
hsa05120	cell signaling in Helicobacter pylori	1/25	0.196655	0.334781	0.251363	1
hsa04520	Adherens junction	1/25	0.199175	0.335082	0.251589	1
hsa01524	Platinum drug resistance	1/25	0.204193	0.339529	0.254928	1
hsa05220	Chronic myeloid leukemia	1/25	0.211663	0.347905	0.261217	1
hsa00980	oolism of xenobiotics by cytochrome	1/25	0.214138	0.347973	0.261268	1
hsa04662	B cell receptor signaling pathway	1/25	0.226401	0.363588	0.272992	1
hsa05204	Chemical carcinogenesis	1/25	0.228832	0.363588	0.272992	1
hsa04010	MAPK signaling pathway	2/25	0.232043	0.364639	0.273781	2
hsa05222	Small cell lung cancer	1/25	0.250379	0.384484	0.288681	1
hsa05206	MicroRNAs in cancer	2/25	0.250663	0.384484	0.288681	2
hsa04912	GnRH signaling pathway	1/25	0.252737	0.384484	0.288681	1
hsa05017	Spinocerebellar ataxia	1/25	0.264422	0.390042	0.292855	1
hsa04914	ogesterone-mediated oocyte maturati	1/25	0.266738	0.390042	0.292855	1
hsa04750	natory mediator regulation of TRP cl	1/25	0.269046	0.390042	0.292855	1
hsa05142	igas disease (American trypanosomia	1/25	0.273643	0.390042	0.292855	1
hsa05165	Human papillomavirus infection	2/25	0.274039	0.390042	0.292855	2
hsa04620	Toll-like receptor signaling pathway	1/25	0.278212	0.390042	0.292855	1
hsa04625	-type lectin receptor signaling pathwa	1/25	0.278212	0.390042	0.292855	1
hsa04660	T cell receptor signaling pathway	1/25	0.278212	0.390042	0.292855	1
hsa04659	Th17 cell differentiation	1/25	0.285013	0.395698	0.297101	1
hsa04668	TNF signaling pathway	1/25	0.296213	0.402602	0.302285	1
hsa05145	Toxoplasmosis	1/25	0.296213	0.402602	0.302285	1
hsa04725	Cholinergic synapse	1/25	0.298433	0.402602	0.302285	1
hsa04151	PI3K-Akt signaling pathway	2/25	0.302112	0.403757	0.303152	2
hsa04071	Sphingolipid signaling pathway	1/25	0.31161	0.404242	0.303517	1
hsa04722	Neurotrophin signaling pathway	1/25	0.31161	0.404242	0.303517	1

hsa04935	th hormone synthesis, secretion and a	1/25	0.31161	0.404242	0.303517	1
hsa05135	Yersinia infection	1/25	0.313783	0.404242	0.303517	1
hsa04611	Platelet activation	1/25	0.322409	0.411647	0.309076	1
hsa00230	Purine metabolism	1/25	0.335153	0.424132	0.31845	1
hsa04210	Apoptosis	1/25	0.347667	0.425201	0.319253	1
hsa04140	Autophagy - animal	1/25	0.349731	0.425201	0.319253	1
hsa04371	Apelin signaling pathway	1/25	0.349731	0.425201	0.319253	1
hsa05162	Measles	1/25	0.351788	0.425201	0.319253	1
hsa04910	Insulin signaling pathway	1/25	0.353839	0.425201	0.319253	1
hsa05418	Fluid shear stress and atherosclerosis	1/25	0.353839	0.425201	0.319253	1
hsa04550	pathways regulating pluripotency of	1/25	0.361981	0.431361	0.323878	1
hsa04723	Retrograde endocannabinoid signalin	1/25	0.37202	0.438388	0.329154	1
hsa04261	drenergic signaling in cardiomyocyte	1/25	0.37401	0.438388	0.329154	1
hsa04921	Oxytocin signaling pathway	1/25	0.383867	0.441379	0.331399	1
hsa04150	mTOR signaling pathway	1/25	0.38582	0.441379	0.331399	1
hsa04934	Cushing syndrome	1/25	0.38582	0.441379	0.331399	1
hsa04218	Cellular senescence	1/25	0.395499	0.445326	0.334363	1
hsa04310	Wnt signaling pathway	1/25	0.395499	0.445326	0.334363	1
hsa05161	Hepatitis B	1/25	0.399329	0.446126	0.334964	1
hsa04022	cGMP-PKG signaling pathway	1/25	0.408803	0.45317	0.340253	1
hsa05164	Influenza A	1/25	0.414419	0.455861	0.342273	1
hsa05152	Tuberculosis	1/25	0.43277	0.472413	0.354701	1
hsa05034	Alcoholism	1/25	0.445285	0.482392	0.362194	1
hsa05169	Epstein-Barr virus infection	1/25	0.469526	0.502867	0.377566	1
hsa05130	Pathogenic Escherichia coli infection	1/25	0.471218	0.502867	0.377566	1
hsa05170	man immunodeficiency virus 1 infect	1/25	0.487855	0.512631	0.384898	1
hsa04810	Regulation of actin cytoskeleton	1/25	0.489491	0.512631	0.384898	1
hsa05132	Salmonella infection	1/25	0.491122	0.512631	0.384898	1
hsa05166	uman T-cell leukemia virus 1 infecti	1/25	0.499201	0.517288	0.388395	1
hsa04144	Endocytosis	1/25	0.542219	0.556833	0.418086	1
hsa05012	Parkinson disease	1/25	0.545152	0.556833	0.418086	1
hsa05016	Huntington disease	1/25	0.576247	0.58442	0.438799	1
hsa05010	Alzheimer disease	1/25	0.65441	0.659018	0.49481	1
hsa05168	Herpes simplex virus 1 infection	1/25	0.793609	0.793609	0.595864	1

Table S4 Up and down-regulated genes in ZSD group compared with Control group

Number	Symbol	Location	Biological function	Fold change	Up/down regulation
1	ACSL4	Xq23	acyl-CoA synthetase long chain family member 4, participating in the process of lipid biosynthesis and fatty acid degradation	2.31	2.31
2	CA9	9p13.3	carbonic anhydrase 9, participating in the process of respiration, calcification, acid-base balance, bone resorption	2.29	2.29
3	RB1	13q14.2	RB transcriptional corepressor 1, regulating cell cycle	2.20	2.20
4	TNFSF10	3q26.31	TNF superfamily member 10, inducing apoptosis in transformed and tumor cells	9.85	9.85
5	BBC3	19q13.32	BCL2 binding component 3, binding to anti-apoptotic Bcl-2 family members to induce mitochondrial dysfunction and caspase activation.	0.22	-4.61
6	BCL2	18q21.33	BCL2 apoptosis regulator, anti-apoptotic protein	0.11	-8.81
7	CCND2	12p13.32	cyclin D2, regulating the cell-cycle during G(1)/S transition	0.39	-2.60
8	MMP7	11q22.2	matrix metallopeptidase 7, breakdown of extracellular matrix	0.37	-2.69
9	WNT6	2q35	Wnt family member 6, regulating cell fate and patterning during embryogenesis	0.28	-3.62
10	HEY1	8q21.13	hes related family bHLH transcription factor with YRPW motif 1, target gene of Notch and c-Jun signal transduction pathways	0.2	-4.05
11	HEYL	1p34.2	hes related family bHLH transcription factor with YRPW motif like, an effector of Notch signaling and a regulator of cell fate decisions	0.39	-2.58
12	ID1	20q11.21	inhibitor of DNA binding 1, HLH protein, regulating cell growth, senescence, and differentiation	0.27	-3.77
13	PTCH1	9q22.32	patched 1, a receptor for the secreted hedgehog ligands	0.45	-2.23

14	BMP2	20p12.3	bone morphogenetic protein 2, binding to TGF-beta receptors, regulating bone and cartilage development	0.40	-2.53
15	CEBPD	8q11.21	CCAAT enhancer binding protein delta, regulating immune and inflammatory responses	0.23	-4.40
16	FCER2	19p13.2	Fc fragment of IgE receptor II, regulating cell growth and differentiation	0.13	-7.89
17	LRG1	19p13.3	leucine rich alpha-2-glycoprotein 1, participate in protein-protein interaction, signal transduction, and cell adhesion and development	0.08	-13.24
18	CPT2	1p32.3	carnitine palmitoyltransferase 2, Participate in long chain fatty acid oxidation	0.11	-8.70
19	GSR	8p12	glutathione-disulfide reductase, regulating cellular antioxidant defense	0.31	-3.20
20	IFRD1	7q31.1	interferon related developmental regulator 1, regulating cell growth and differentiation	0.27	-3.67
